# Circular oligomeric particles formed by Ros/MucR family members mediate DNA organization in α-proteobacteria

**DOI:** 10.1093/nar/gkae1104

**Published:** 2024-11-26

**Authors:** Antonio Chaves-Sanjuan, Gianluca D’Abrosca, Veronica Russo, Bert van Erp, Alessandro Del Cont-Bernard, Riccardo Capelli, Luciano Pirone, Martina Slapakova, Domenico Sgambati, Roberto Fattorusso, Carla Isernia, Luigi Russo, Ian S Barton, Roy Martin Roop, Emilia M Pedone, Martino Bolognesi, Remus T Dame, Paolo V Pedone, Marco Nardini, Gaetano Malgieri, Ilaria Baglivo

**Affiliations:** Department of Biosciences, University of Milan, Via Celoria 26, 20133 Milano, Italy; Fondazione Romeo e Enrica Invernizzi and NOLIMITS, University of Milan, Via Celoria 26, 20133 Milan, Italy; Department of Clinical and Experimental Medicine, University of Foggia, Viale Pinto 1, 71100 Foggia, Italy; Department of Environmental, Biological, Pharmaceutical Sciences and Technologies, University of Campania “Luigi Vanvitelli”, Via Vivaldi 43, 81100 Caserta, Italy; Macromolecular Biochemistry, Leiden Institute of Chemistry, Leiden University, Einsteinweg 55, Leiden 2333CC, The Netherlands; Centre for Microbial Cell Biology, Leiden University, Einsteinweg 55, Leiden 2333CC, The Netherlands; Centre for Interdisciplinary Genome Research, Leiden University, Einsteinweg 55, Leiden 2333CC, The Netherlands; Department of Biosciences, University of Milan, Via Celoria 26, 20133 Milano, Italy; Department of Biosciences, University of Milan, Via Celoria 26, 20133 Milano, Italy; Institute of Biostructures and Bioimaging, CNR, Via Pietro Castellino 111, 80131 Naples, Italy; Department of Environmental, Biological, Pharmaceutical Sciences and Technologies, University of Campania “Luigi Vanvitelli”, Via Vivaldi 43, 81100 Caserta, Italy; Department of Environmental, Biological, Pharmaceutical Sciences and Technologies, University of Campania “Luigi Vanvitelli”, Via Vivaldi 43, 81100 Caserta, Italy; Department of Environmental, Biological, Pharmaceutical Sciences and Technologies, University of Campania “Luigi Vanvitelli”, Via Vivaldi 43, 81100 Caserta, Italy; Department of Environmental, Biological, Pharmaceutical Sciences and Technologies, University of Campania “Luigi Vanvitelli”, Via Vivaldi 43, 81100 Caserta, Italy; Department of Environmental, Biological, Pharmaceutical Sciences and Technologies, University of Campania “Luigi Vanvitelli”, Via Vivaldi 43, 81100 Caserta, Italy; Department of Microbiology and Immunology, Brody School of Medicine, East Carolina University, 600 Moye Blvd, Greenville, NC 27834, USA; Department of Microbiology and Immunology, Brody School of Medicine, East Carolina University, 600 Moye Blvd, Greenville, NC 27834, USA; Institute of Biostructures and Bioimaging, CNR, Via Pietro Castellino 111, 80131 Naples, Italy; Department of Biosciences, University of Milan, Via Celoria 26, 20133 Milano, Italy; Fondazione Romeo e Enrica Invernizzi and NOLIMITS, University of Milan, Via Celoria 26, 20133 Milan, Italy; Macromolecular Biochemistry, Leiden Institute of Chemistry, Leiden University, Einsteinweg 55, Leiden 2333CC, The Netherlands; Centre for Microbial Cell Biology, Leiden University, Einsteinweg 55, Leiden 2333CC, The Netherlands; Centre for Interdisciplinary Genome Research, Leiden University, Einsteinweg 55, Leiden 2333CC, The Netherlands; Department of Environmental, Biological, Pharmaceutical Sciences and Technologies, University of Campania “Luigi Vanvitelli”, Via Vivaldi 43, 81100 Caserta, Italy; Department of Biosciences, University of Milan, Via Celoria 26, 20133 Milano, Italy; Fondazione Romeo e Enrica Invernizzi and NOLIMITS, University of Milan, Via Celoria 26, 20133 Milan, Italy; Department of Environmental, Biological, Pharmaceutical Sciences and Technologies, University of Campania “Luigi Vanvitelli”, Via Vivaldi 43, 81100 Caserta, Italy; Department of Environmental, Biological, Pharmaceutical Sciences and Technologies, University of Campania “Luigi Vanvitelli”, Via Vivaldi 43, 81100 Caserta, Italy

## Abstract

The transcriptional regulator MucR from *Brucella* species controls the expression of many genes, including those involved in virulence, by binding AT-rich DNA regions. MucR and its homologs belong to the Ros/MucR family, whose members occur in α-proteobacteria. MucR is a recent addition to the family of histone-like nucleoid structuring (H-NS) proteins. Indeed, despite the lack of sequence homology, MucR bears many functional similarities with H-NS and H-NS-like proteins, structuring the bacterial genome and acting as global regulators of transcription. Here we present an integrated cryogenic electron microscopy (cryo-EM), nuclear magnetic resonance, modeling and biochemical study shedding light on the functional architecture of MucR from *Brucella abortus* and its homolog Ml5 from *Mesorhizobium loti*. We show that MucR and Ml5 fold in a circular quaternary assembly, which allows it to bridge and condense DNA by binding AT-rich sequences. Our results show that Ros/MucR family members are a novel type of H-NS-like proteins and, based on previous studies, provide a model connecting nucleoid structure and transcription regulation in α-proteobacteria.

## Graphical Abstract

**Graphical Abstract:**
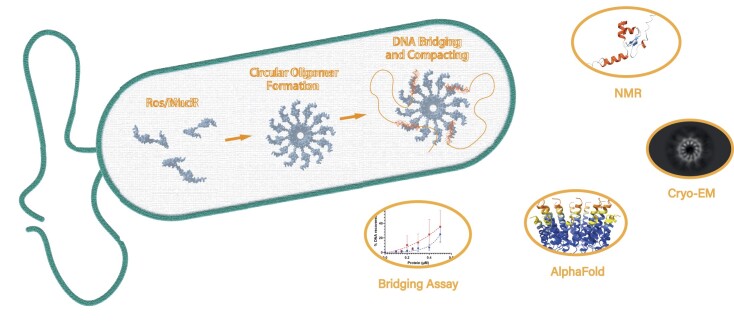

## Introduction

Nucleoid-associated proteins (NAPs) are known to organize and fold bacterial genomic DNA in a dynamic chromosome structure that is responsive to environmental changes (1–3). Among the best-studied NAPs are the histone-like nucleoid structuring (H-NS) proteins, originally discovered in *Escherichia coli* (4), which play a crucial role in local genome organization and in gene expression regulation (5,6). In particular, H-NS proteins repress genes involved in bacterial virulence acquired by Horizontal Gene Transfer (HGT), thus acting as ‘xenogeneic silencers’ (7). The H-NS family has grown over the years and includes H-NS-like proteins identified in many bacterial species (8–10), such as *Mycobacteria* sp., *Pseudomonas* sp., *Bacillus* sp. (11–13). H-NS and H-NS-like proteins, despite low sequence homology, share a common tertiary structure organization, consisting of an oligomerization domain at the N-terminus (NTD) and a DNA-binding domain (DBD) at the C-terminus (2) as well as the ability to bind and structure DNA in an environment-modulated manner (5,6,8,14).

Classical H-NS and H-NS-like proteins assemble by means of head–head and tail–tail interactions into high-order oligomers, forming filaments that bind across AT-rich regions on the genome through their DBDs (15–19). The presence of multiple DBDs in H-NS filaments confers structural ‘multivalency’, which allows concurrent binding to DNA and bridging more than one DNA duplex, thus forming DNA:H-NS:DNA complexes (16,20,21). DNA-bridging activity is tuned by osmolarity, temperature and other environmental stimuli that affect the structure of bridged H-NS filaments, facilitating transcription (1,3,20,22,23). The capacity to bridge DNA is fundamental for organizing the bacterial genome and regulating gene expression.

H-NS and H-NS-like proteins have been identified primarily in β- and γ-proteobacteria. However, H-NS-like proteins are not detected in the majority of α-proteobacteria (9,10).

MucR from α-proteobacterium *Brucella abortus* forms high-order oligomers through the NTD and binds preferentially AT-rich DNA targets containing TpA steps through its C-terminal DBD (24–28). Chromosome Conformation Capture experiments have also demonstrated that MucR plays an important role in genome organization in *B. abortus* (29). Furthermore, its homolog from *Sinorhizobium fredii*, was shown to bridge DNA *in vitro* and was recognized as a xenogeneic silencer because of its role in expression regulation of genes acquired by HGT (27,30,31). These characteristics establish MucR as an H-NS-like protein (8).

MucR homologs, also named Ros in some bacterial species, have been identified in several α-proteobacteria such as *Agrobacterium tumefaciens*, *Rhizobia* sp., *Caulabcter crescentus*. These proteins, constituting the Ros/MucR family, have been characterized as transcriptional regulators controlling expression of genes required for successful infection of, or symbiosis with, eukaryotic hosts (32–35). Ros/MucR proteins constitutively repress these genes and their activation occurs only under particular environmental conditions. In *A. tumefaciens*, Ros represses its own gene as well as *virC*, *virD* and *ipt* virulence genes, by binding to their promoters (32,36–38). In *Rhizobia* species, MucR represses its own gene and controls the expression of genes required for exopolysaccharide synthesis, chemotaxis, motility and nodulation (32,39,40).

Only limited structural data are currently available for Ros/MucR family members. The structure of the *A. tumefaciens* Ros DBD was solved via nuclear magnetic resonance (NMR); the domain shows a βββαα architecture that coordinates a zinc ion, forming a zinc-binding domain (41). Studies of the homologous proteins show that the zinc coordination sphere may vary, an Asp residue often replacing the more classical Cys and/or His residues. Furthermore, some of the homologs, such as *Mesorhizobium loti* Ml5, display a zinc-free DBD in which the first coordinating Cys is substituted by a serine (30,42). To date, among Ros/MucR family members, only *B. abortus* MucR has been recognized as an H-NS-like protein (8,29,30). However, information on the protein quaternary assembly and DNA compaction properties in this family is lacking.

Here, we report the structural characterization of the zinc-bound *B. abortus* MucR, and the zinc-free *M. loti* Ml5 as representative members of the Ros/MucR protein family. Single particle cryo-EM and NMR data are integrated with *in silico* models to describe the higher-order oligomers formed by the two proteins. We show that both MucR and Ml5 act as H-NS-like proteins and their circular oligomers, although different from typical H-NS filaments, they still facilitate *in trans* interactions that are potentially involved in spatial genome organization and gene regulation. Finally, we propose a model for DNA organization by oligomeric MucR and Ml5 proteins. Structural and functional similarities shared by MucR, Ml5 and their homologs, provide support for the Ros/MucR family as a distinct sub-family of H-NS-like proteins.

## Materials and methods

### Cloning, protein expression and purification

The DNA coding sequence for Ml5 (UniprotKB, protein accession number: Q98A76; gene name mll6119) was amplified by polymerase chain reaction (PCR) using *M. loti* genomic DNA, extracted by ULTRAprep Genomic DNA kit (miniprep genomic DNA BAC – Top line), as a template. Primer sequences designed on the base of the *ml5* gene sequence are reported in Supplementary Table S1 (primer 1 and 2).

The PCR product was purified from agarose gel by using gel extraction kit (Qiagen). Then, the purified DNA fragment and the pET-22b(+) vector were digested with *NdeI* and *EcoRI*. After digestion, both the fragment and the linearized vector were purified from agarose gel and then used for a ligation reaction to obtain *ml5*-pET-22B(+).

Site directed mutagenesis using PCR was performed to generate the sequence encoding for Ml5^L34L37I38A^. Primers to introduce the required mutations were designed (Supplementary Table S1, primers 3 and 4) and employed for PCR using *ml*5- pET-22b(+) as template. Following the same procedure as described above, the PCR fragment was gel purified, double digested with *NdeI* and *EcoRI*, purified again from agarose gel after digestion and finally ligated into pET-22b(+) to obtain *ml5*^L34L37I38A^- pET-22b(+).

The plasmids used for expression of *B. abortus* MucR and the mutant MucR^L36L39I40A^ were described in Baglivo *et al.* (24) and Pirone *et al.* (26).

Deletion mutants corresponding to the DBD regions were produced using the plasmid clones reported in Baglivo *et al.* (Ml5_56–154_) (42) and in Pirone *et al.* (MucR_57–142_) (26).

To express the proteins used for this study, *E. coli* BL21(DE3) strain was transformed with the plasmid pET-22b(+) containing the coding sequences for *B. abortus* MucR, MucR^L36L39I40A^, *M. loti* Ml5 or Ml5^L34L37I38A^. The transformed *E. coli* Bl21(DE3) strain was cultured in Luria-Bertani (LB) at 37°C while shaking (200 rpm) until reaching an optical density at 600 nm of 0.4. Next, bacterial cultures were chilled on ice for 10 min to prevent further growth of bacteria before inducing protein expression. IPTG (Isopropyl-ß-D-thiogalactopyranoside) was added to the chilled cultures at a final concentration of 1 mM to induce protein expression, which was carried out by incubation of cultures while shaking (200 rpm) at 28°C. Bacterial cells were harvested after 1 h from the induction.

The truncated proteins studied by NMR, MucR_57–142_ and Ml5_56–154_, consisting of the DBDs, were expressed following the method reported above, but using minimal medium containing 0.5 g/l ^15^NH_4_Cl as the only nitrogen source.

Purification of proteins was carried out by cation exchange chromatography, followed by gel filtration, as previously reported (42).

Proteins eluted from the Mono S HR 5/5 cation exchange chromatography column (GE HealthCare) in 0.3 M NaCl (MucR^L36L39I40A^ and Ml5^L34L37I38A^), 0.6 M NaCl (Ml5), 0.8 M NaCl (MucR). Gel filtration chromatography to purify Ml5 and MucR was performed using a Superdex S200 column (GE HealthCare) equilibrated in 25 mM Tris (pH = 7.0), 0.6 M NaCl. Gel filtration of the protein mutants MucR^L36L39I40A^ and Ml5^L34L37I38A^ was carried out using a Superdex S200 column (GE HealthCare) equilibrated in 25 mM Tris (pH = 7.0), 0.3 M NaCl. Gel filtration chromatography of Ml5_56–149_ and MucR_57–142_ was performed by using a Superdex 75 column (GE HealthCare) equilibrated in 25 mM Tris (pH = 7.0), 0.3 M NaCl.

Proteins purified for NMR analyses were concentrated after purification by using amicon ultra centrifugal filters (cut off 3k Da) following the manufacturer's protocol.

### Cryogenic electron microscopy (cryo-EM) sample preparation, data collection and data processing

Full-length MucR at a concentration of 1 mg/ml in 25 mM Tris (pH = 7.0), 0.6 M NaCl buffer was diluted to a final concentration of 0.66 mg/ml and 25 mM Tris (pH = 7.0), 0.4 M NaCl buffer.

MucR sample vitrification was carried out with a Mark IV Vitrobot (Thermo Fisher Scientific); 3 μl of MucR sample were applied to a Quantifoil R 1.2/1.3 Cu 300-mesh grid previously glow-discharged at 30 mA for 30 s in a GloQube (Quorum Technologies). The sample was incubated on grid for 60 s at 4 °C and 100% humidity, blotted and plunge-frozen into liquid ethane. For experiments with DNA, MucR was mixed with 60 bp double stranded oligonucleotide babR60 (43), a 232 bp DNA fragment containing the *mucR* promoter (33) or linearized pGEM-3Z in a 45:1 ratio in 0.1 M KCl, 50 mM Tris (pH = 8.0), 12.5 mM MgCl_2_ buffer. MucR/DNA vitrification was carried out as for MucR samples.

Vitrified grids were transferred to a Talos Arctica (Thermo Fisher Scientific) operated at 200 kV and equipped with a Falcon 3 direct electron detector (Thermo Fisher Scientific). MucR/DNA was imaged at 73000 ×. MucR vitrified sample was used for automatic data collection. A total of 4˙020 movies were collected using EPU 2.8 (Thermo Fisher Scientific) in electron counting mode with an applied dose of 60 e^−^/Å^2^ divided in 60 frames at a magnification of 120k×, that corresponds to a pixel size of 0.889 Å/pixel, and a defocus range of −0.8 to −2.5 μm.

Movies were preprocessed with WARP 1.0.9 (44). A 5 × 5 × 60 model was used for motion correction using a 35–7 Å resolution range weighted with a -500 Å^2^ B factor. CTF estimation was done using a 40–3.5 Å resolution range and a 5 × 5 model. Particle picking was performed using as model the deep convolutional neural network BoxNet2Mask_20 180 918 specifically trained with four manually picked micrographs, resulting in 2˙387˙280 particles that were extracted with a box size of 260 × 260 pixels and a pixel size of 0.889, inverted, normalized and imported into cryosparc 4.2.1 (45) for further processing. Three rounds of 2D classification with 120 Å mask were used to select a homogeneous set of 179˙035 particles showing features of 12-protomer closed-circle particles. An *ab initio* reconstruction with a target resolution of 7 Å, an increased batch size of 600 particles and C12 symmetry was used to generate an initial model and obtain a first particle alignment. Particles were finally refined with a local refinement using C12 symmetry and a static mask generated from the *ab initio* reconstruction low-pass filtered to 15 Å, threshold 0.2, dilation radius of 2 pixels and a soft padding width of 10 pixels (Supplementary Figure S1A). The final resolution of the reconstruction was 3.8 Å resolution according to an FSC (Fourier Shell Correlation) cut-off of 0.143 (Supplementary Figure S1B). The final reconstruction was low-pass filtered to 8 Å.

### Light scattering

To measure molecular weight of Ml5 and Ml5^L34L37I38A^ a MiniDAWN Treos spectrometer (Wyatt Instrument Technology Corp.) equipped with a laser operating at 658 nm was used connected in-line to a size-exclusion chromatography column. Samples at a concentration of 1 mg/ml were loaded onto a Superdex 200 column (10 × 30 cm, GEHealthcare) equilibrated in 25 mM Tris (pH = 7.0), 0.6 M NaCl for Ml5 and 25 mM Tris (pH = 7.0), 0.3 M NaCl for Ml5^L34L37I38A^ and connected to a triple-angle light scattering (LS) detector equipped with a QELS (Quasi-Elastic Light Scattering) module. A constant flow rate of 0.5 ml/min was applied. Elution profiles were detected by a Shodex interferometric refractometer and a mini Dawn TREOS LS system. The Astra 5.3.4.14 software (Wyatt Technology) was used to analyze data. Duplicate of each experiment were carried out.

### AlphaFold2 prediction and MucR model fitting onto the cryo-EM density map

The models and the complexes of this study were predicted using AlphaFold2 and AlphaFold2-Multimer algorithm within the ColabFold notebooks (46,47). The default settings with Amber relaxation were employed. For predicting complex structures, amino acids primary sequences of the N-terminal regions (1–56 of MucR and 1–55 of Ml5) of the two proteins were used as input 12 times, according to the cryo-EM data. The five models obtained were verified for consistency and the top-ranked model was selected in each case. The accuracy of an AlphaFold2 (AF2) prediction is indicated by pLDDT score and pTM score, which rank monomeric models and protein–protein complexes, respectively. In Supplementary Table S2 the confidence metrics for the predicted complexes are summarized.

The N-terminal complex model of 12-mer MucR generated by AF2 was manually fitted onto the cryo-EM density map using ChimeraX (48) software; the fitting was then refined using the ‘fit to map’ command, independently on the two helices of each monomer, to take in consideration the flexibility of the connecting loop.

### NMR data acquisition and chemical shifts analysis

The NMR experiments were acquired at 298K on a Bruker Avance III HD 600 MHz spectrometer, equipped with a triple resonance Prodigy N2 cryoprobe with a z-axis pulse field gradient. The NMR samples of MucR_57–142_ and Ml5_56–154_ contained 200 and 500 μM, respectively, of purified ^15^N labeled proteins in a 20 mM phosphate buffer, 0.3 M NaCl at pH = 6.8.

A standard set of triple resonance experiments (3D HNCA, 3D CBCANH, 3D CBCA(CO)NH, were performed for MucR_57–142_, leading to the assignment of the backbone resonances of Cα, Cβ, N and HN. All NMR data were processed by means of Bruker software Topspin 4.0.8 and the spectra were analyzed using CARA (downloaded from cara.nmr.ch) and SPARKY software (49).

ΔδCα and ΔδCβ were obtained according to the random coil chemical shifts values proposed by Whishart *et al.*(50). The back-calculated chemical shifts for AF2 MucR_57-142_ best model were obtained by means of Shiftx2 server (http://www.shiftx2.ca/). The quality Q-factor was calculated following the equation: Q = rms(CS^obs^ − CS^pred^)/rms(CS^obs^), where CS^obs^ and CS^pred^ are the measured and back calculated chemical shifts, respectively.

The ^15^N transverse relaxation rates (R_2_) assessed from ^15^N linewidths in the [^1^H-^15^N] HSQC spectra were compared with the relaxation parameters predicted from the AF2 model using HYDRONMR (51).

All structures were visualized and analyzed using the software MOLMOL (52), PyMol (DeLano Scientific, San Carlos, California, USA), and ChimeraX (48).

### MucR_57-142_–DNA docking

A standard B–DNA conformation has been built by 3DNA software (53) using the DNA sequence:

(5′→3′ strand ATGAAGTTATATTCAATATAAAAGTAGAAT)

The program HADDOCK (54) was used to dock MucR_57–142_ to the DNA using solvent-accessible residues defined by DISPLAR (55) and ambiguous intermolecular restraints defined according to established criteria. HADDOCK software can make use of an array of ambiguous intermolecular restraints (AIRs), including those derived from biochemical and biophysical data.

Residues located on the binding surface of MucR_57–142_ which showed a relative solvent accessibility above 50% were defined as ‘active residues’, while all the residues close to the active ones and exhibiting a relative accessibility lower than 50% were defined as ‘passive’. The model DNA structure, MucR_57–142_ AF2 model and the intermolecular restraint were used as input to HADDOCK that generated 200 solutions which were sorted into 18 clusters using a pairwise backbone r.m.s.d. (root-mean-square deviation) of 7.5 Å as cut-off criterion. Clusters were ordered on the basis of the calculated intermolecular energy according to their HADDOCK scores. Structures were also clustered considering their Z-scores that indicate the standard deviations from the average of a particular cluster in terms of HADDOCK score. The top two clusters had HADDOCK scores of −69.5 ± 16.0 and −64.7 ± 11.0, and Z scores of −1.9 and −1.2, respectively. From cluster 1, the best structure with the lowest HADDOCK score was assumed as the representative model of the MucR_57–142_–DNA complex, also on the basis of literature data (24,27,56).

### Normal mode simulation

The Normal Mode-based Simulation (NMSim) methodology (57) has been used as an alternative to the conventional MD (Molecular Dynamics) simulation. Starting from the predicted AF2 models of MucR and Ml5 NTDs, we generated an ensemble of 2500 conformers with NMSim using the following parameters suitable for sampling large-scale motions: E-cutoff for H-bonds (kcal/mol) = −1; hydrophobic cutoff (Å) = 0.35; hydrophobic method = 3; RCNMA NM-method = RCNMA; cutoff for C-alpha atoms (Å) = 10; NMSim number of trajectory = 5; number of NMSim cycles = 1; side-chain distorsions = 0.05; number of simulation cycles = 500; output frequency = 1; NM modes range = 1–5; ROG mode = none; step size = 0.

### Ultraviolet-vis spectroscopy

The native zinc ion was removed to obtain apo-MucR_57–142_ by lowering the pH to 2.5 by adding 0.1 M HCl until reaching the required pH value in the presence of 150 μM TCEP (Tris(2-carboxyethyl)phosphine). The sample was then dialyzed against 10 mM Tris, 150 μM TCEP (pH 2.5) to remove zinc from the solution. The pH was finally readjusted to 6.5 by adding small aliquots of 0.1M HCl and kept at 6.5 throughout the experiments. Protein concentrations were obtained using absorption at 280 nm at pH 2.5. Spectra for the Co(II) addition experiments to 4 μM apo-MucR_57–142_ were recorded in 10 mM Tris, 20 μM TCEP (pH = 6.5), on a Shimadzu UV-1800 spectrophotometer in the range of 200–800 nm at room temperature. The apo-protein solution was titrated with aliquots of 0.1 mM CoCl_2_ solution up to 1.6 Co(II)/protein ratio. Each addition corresponded to an increase of 0.4 μM of final Co(II) concentration in solution. Experiments were performed in triplicate; the average of three independent measurements is reported.

The change in absorbance at 340 nm, indicative of the LCMT (ligand-to-metal charge transfer) transition (58), was used to calculate the Co(II) binding affinity constant. To estimate the Zn(II) binding affinity constant, a reverse titration experiment was performed: ZnCl_2_ solution (1.0 mM) was added stepwise to the Co(II)-apo-MucR_57–142_ complex up to a Zn/apoRos87 ratio of 1.5. The titration curves were fitted as reported previously (59).

### Circular Dichroism

Circular Dichroism (CD) experiments were collected using a JASCO J-815 spectropolarimeter equipped with a Peltier temperature control. Data were collected in the 200–260 nm wavelength range using a quartz cuvette with a 1 cm pathlength, with a scanning speed of 50 nm/min, a data pitch of 1 nm, a band width of 1 nm. All CD samples contained ∼15 μM of proteins in 10 mM Tris and 150 μM TCEP at pH 6.5. All the spectra were acquired in triplicate and the background signal of buffer alone was subtracted. Secondary structure content was estimated using DichroWeb server (60).

### Electrophoretic mobility shift assay

Electrophoretic Mobility Shift Assays (EMSAs) were carried out as previously reported (61,62). In short, 0.35 pmol of linearized pGEM-3Z (Promega) plasmid or 5 pmol of babR60 double-stranded oligonucleotide (43) was used as a target for protein binding. Varying amounts of proteins were used as indicated in the text of the results section. Proteins and DNA targets were mixed in a buffer containing 25 mM HEPES (pH = 7.9), 50 mM KCl, 6.25 mM MgCl_2_, 5% glycerol. Samples were incubated 10 min on ice and loaded onto a 5% polyacrylamide gel or onto a 1% agarose gel for samples containing linearized plasmid as DNA target.

Electrophoresis of samples loaded onto 5% polyacrylamide gel was performed in 0.5X TBE at room temperature for 70 min at 200 V. Gels were stained 20 min using Diamond™ Nucleic Acid Dye (Promega) following the manufacturer’s instructions and imaged using a Typhoon Trio + scanner (GE Healthcare).

Electrophoresis of samples loaded onto agarose gel was carried out in 1× TBE at room temperature for 1 h at 100 V. Gels were stained by Ethidium bromide solution and analyzed by ultraviolet transilluminator or using Typhoon Trio + scanner (GE Healthcare).

The results of EMSAs shown in this study are representative of >5 replicates.

### Bridging assay

The *mucR* promoter sequence (25,33) and a sequence derived from the *babR* promoter, called babR60 (43), were used to design the probes (Supplementary Table S1) for the bridging experiments. The DNA sequences chosen as targets were first cloned into pBTH154 (63). The *mucR* promoter was cloned into *MauBI* and *ApaI* restriction sites, while the babR60 oligonucleotide was extended 26 bp, taken from the beginning of babR60 sequence, to be cloned into *AleI* and *BstAPI* restriction sites, thus generating pBTH154 containing the *B. abortus mucR* promoter (plasmid vector pRD485) or babR60 sequence (plasmid vector pRD486). The probes were generated by PCR using pRD485 or pRD486 as templates with primers 5 and 6 (Supplementary Table S1) for prey DNA and using primer 6 and a 5′ biotinylated variant of primer 5 for bait DNA. The PCR products were purified using a GeneElute kit (Sigma Aldrich) and ^32^P labeled with Polynucleotide Kinase and γ-^32^P ATP (64).

For each bridging assay, 6 μl of streptavidin-coated paramagnetic beads was washed with 50 μl of PBS [12 mM NaPO_4_ (pH = 7.4), 137 mM NaCl], then washed twice with 50 μl of CB [20 mM Tris–HCl (pH = 8.0), 2 mM EDTA, 2 M NaCl, 2 mg/ml Acetylated BSA, 0.04% Tween20] and then resuspended in 6 μl of CB. Next, 100 pmol (in a total volume of 3 μl) of biotinylated DNA was added into half of the suspension and incubated with the beads at 25°C for 20 min in an Eppendorf shaker at 1000 rpm while the other half of the bead suspension was incubated without DNA as a control. After incubation the beads were washed twice with 16 μl of IB [100 mM Tris–HCl (pH = 8.0), 0.2% Tween20, 10 mg/ml acetylated BSA and 400 mM KCl or 380 mM KCl for MucR WT or Ml5 WT, respectively] and resuspended in 16 μl of IB; 2 μl of protein (MucR wt, Ml5 WT, MucR^L36L39I40A^ or Ml5^L34L37I38A^) and 2 μl of radiolabeled DNA probes (with a minimum of 5000 counts per minute) was added to both bead suspensions, gently mixed and incubated at 25°C for 20 min in the Eppendorf shaker at 1000 rpm. After incubation the beads were gently washed with 20 μl experimental buffer, resuspended in Stop buffer [10 mM Tris–HCl (pH = 8.0), 1 mM EDTA, 200 mM NaCl, 0.2% sodium dodecyl sulfate] and then the sample was transferred to a liquid Cherenkov-scintillation counter to quantify the DNA bridging efficiency.

## Results

### MucR oligomerizes in closed-circular and open-horseshoe quaternary structures

**Figure 1.**
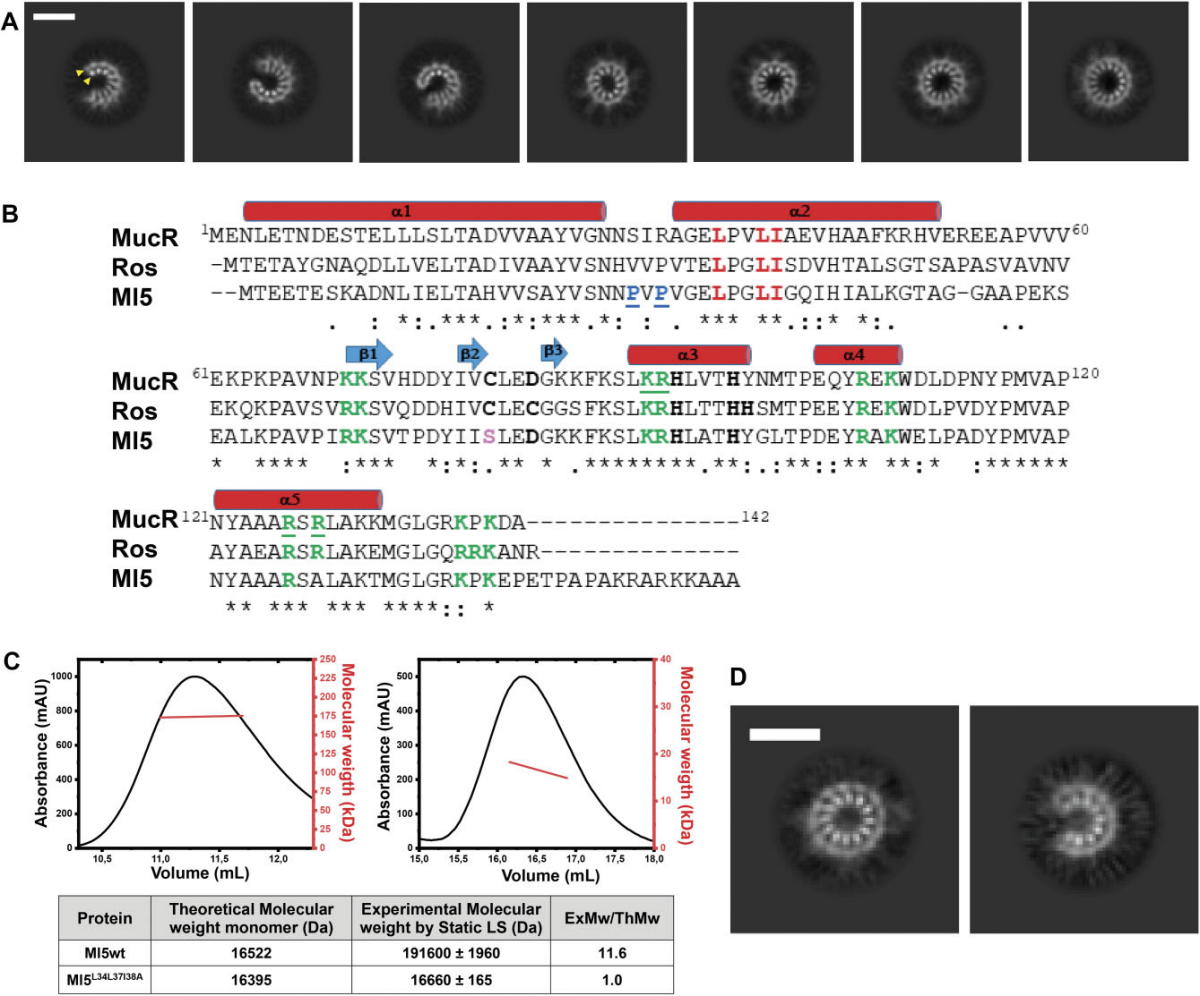
(**A**)**MucR particles**. Representative cryo-EM 2D averages of the full length MucR. Top view identifying from left to right, particles of 9-, 10- and 11-protomer horseshoes and 11-, 12-, 13- and 14-protomers closed-circle particles. The scale bar inserted in the first class represents 6 nm. The signals for the inner and outer helices were marked with a yellow triangle. (**B**) **ClustalW alignment of *B. abortus* MucR, *M. loti* Ml5 and *A. tumefaciens* Ros**. MucR secondary structure elements are indicated. The two proline residues in the linker between the two N-terminal α-helices in Ml5 (see the ‘Results’ section) are underlined blue. Residues coordinating the Zn (II) atom in Ros and MucR, as well as those substituting these coordinating residues in the zinc-free homolog Ml5, are in bold (42). In Ml5, the serine residue substituting the first cysteine coordinating zinc in Ros, is in pink. In Ros, both histidine residues, alternatively functioning as fourth zinc coordinating position, are also highlighted in bold (66). The three residues crucial for oligomerization are in red. Basic regions playing a crucial role in Ros DNA binding and conserved in the homologous proteins are in green. Residues involved in MucR–DNA interaction reported in this study are underlined green. (**C**)**LS analyses of Ml5 (left) and Ml5^L34L37I38A^ (right)**. LS data are reported in the table. The theoretical molecular weight of the monomeric proteins was calculated by ProtParam tool (http://web.expasy.org/protparam/). (**D**)**Ml5 particles**. Representative cryo-EM 2D averages of the full length Ml5. On the left 12-protomer closed-circle particle and on the right, 9-protomer horseshoe particle. The scale bar inserted in the first class represents 6 nm.

Full-length MucR can oligomerize in solution yielding assemblies of different sizes (24,26,65). To gain insights into the stoichiometry and structure of MucR oligomers, we examined the cryo-EM 2D averaged images of the full-length recombinant protein that revealed particles of ∼6–7 nm in diameter, characterized by a repetitive pattern of secondary structure elements compatible with a double layer of inner and outer α-helices (Figure 1A, and Supplementary Figures S1 and S2). Strikingly, MucR particles occur in a variety of multimeric states including (i) closed circular oligomers (formed by 11, 12, 13 and 14 protomers), and (ii) open horseshoe-shaped oligomers (formed by 9, 10 and 11 protomers), the most represented particles being characterized by a closed 12-protomer and 10-protomer assembly. Previous sequence-based predictions indicated that MucR oligomerization is mediated by protein–protein interactions involving the NTD. Substitution studies identified Leu36, Leu39 and Ile40 as residues essential for oligomeric assembly (26,27). MucR particles observed in cryo-EM are compatible with the packing of NTDs in various oligomerization states. This leads us to hypothesize that the protein region linking the NTD to the DBD is flexible. As a result, while the NTD core of the oligomer is rigidly packed and easily detected (albeit variable in composition number), the flexible linker and the DBD structures may have been averaged out in the cryo-EM 2D analysis due to overall conformational heterogeneity.

To further test the above hypothesis regarding the NTD multimerization, cryo-EM experiments under the same conditions were carried out on the MucR^L36L39I40A^ variant, which hosts substitutions in the NTD residues known to hamper MucR oligomerization without affecting the folding of DBD (26,67). Analysis of the collected raw-micrographs clearly revealed the presence of a low number of circular particles (Supplementary Figure S3A), showing that MucR oligomerization was severely impaired in the variant, and that the helix double layer observed in wild-type MucR is the *bona fide* assembly unit corresponding to the NTD oligomerization domains. Thus, our data shed light on the NTD-mediated assembly mechanism of MucR, showing that the oligomerization architecture is variable in terms of number of protomers and type of quaternary assembly (closed-circular and open-horseshoe).

### The zinc-free Ml5 shares the MucR oligomerization mechanism

We analyzed whether the *B. abortus* MucR oligomerization mode might be shared by other members of Ros/MucR family by extending our study to the homologous *M. loti* Ml5. We selected the Ml5 protein because it is the most divergent member of the family, lacking the zinc ion in its DBD (Figure 1B). Previous LS analysis with MucR, and Ml1 and Ml2 from *M. loti*, showed that they all form high-molecular weight oligomers in solution (24). Similarly, LS experiments with Ml5 show a molecular weight of 191.6 ± 2.0 kDa, compatible with a dodecameric assembly (Figure 1C). Based on sequence alignment between the N-terminal region (residues from 1 to 56) of Ml5 and MucR (57% sequence identity) (Figure 1B and Supplementary Figure S4), we produced the Ml5^L34L37I38A^ variant, analogues to MucR^L36L39I40A^, the triple substitution variant that is unable to oligomerize (26). LS analysis revealed that the molecular weight of Ml5^L34L37I38A^ matches that of a monomeric species, confirming that Ml5 oligomerization is effectively hampered by these substitutions (Figure 1C).

We then investigated Ml5 assembly by cryo-EM (Figure 1D and Supplementary Figure S3B). Analysis of 2D averages revealed that, similar to MucR, wild-type Ml5 forms circular and horseshoe-shaped particles. Unlike MucR, however, almost all the particles assembled homogeneously as either 12-protomer closed-circular units or as 9-protomer horseshoe-shaped particles. We further verified that the observed oligomers assemble through the NTD of Ml5 by analyzing cryo-EM images of the Ml5^L34L37I38A^ variant. In fact, the substitutions completely abolished formation of the characteristic circular and horseshoe-shaped particles detected with wild-type Ml5 (Supplementary Figure S3B). Our results show that Ml5 shares the same oligomerization mode with MucR, that requires conserved residues from the NTD (Figure 1B). Based on the ability of several members of the Ros/MucR family to oligomerize, and on the high sequence identity in their NTD regions (Figure 1B), we conclude that the oligomerization mechanism here described for MucR and Ml5 represents a hallmark for all Ros/MucR family members.

### Hydrophobic interactions trigger oligomerization in MucR and Ml5

**Figure 2.**
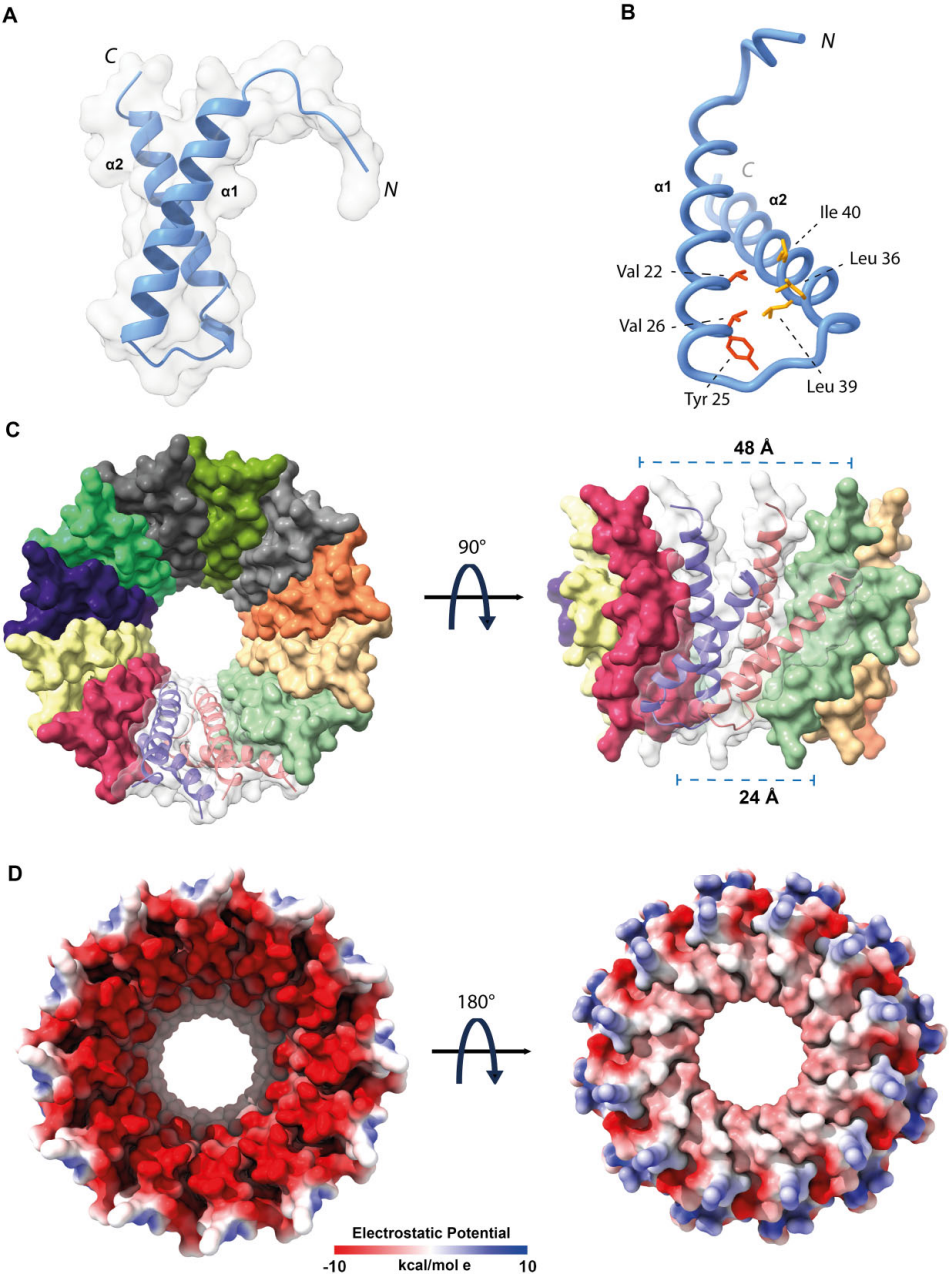
(**A**)**MucR NTD**. AF2 model of MucR NTD (MucR_1–56_). (**B**)**Hydrophobic interactions**. Hydrophobic residues stabilize packing of MucR_1–56_ helices. Sidechains of Leu36, Leu39 and Ile40, crucial for NTD oligomerization, are depicted in orange, while sidechains of Val22, Tyr25 and Val26, necessary for the hydrophobic packing are in red. (**C**)**MucR NTD complex**. Surface representation of AF2 model of the MucR NTD dodecameric complex. Each protomer is depicted with a different color. The secondary structure elements of two protomers are presented as ribbons. (**D**)**Electrostatic potential**. Top and bottom view of the electrostatic potential surface of MucR NTD dodecameric complex.

To further explore the molecular details of the NTD-based oligomerization mechanism, we integrated the cryo-EM data with AI-based structure predictions. We generated a 3D model for the isolated MucR NTD using AlphaFold2 (AF2) (46,47) (Figure 2A). The model of the oligomerization domain highlights two packed antiparallel α-helices encompassing residues Asn3-Asn28 (α1) and Ala33-Glu55 (α2), respectively, which is in agreement with previous data regarding from *S. fredii* MucR (27). Packing of the α1/α2 antiparallel helices is stabilized by a hydrophobic zipper at their contact interface (Supplementary Figure S5), yielding a mutual relative tilt of the helices of ~ 46°. We note that MucR α2 residues Leu36, Leu39 and Ile40 (Leu34, Leu37, Ile38 in Ml5), key to oligomer formation, fall at the inter-helical contact interface (Figure 2B). Thus, they appear to be involved in NTD stabilization within each protomer leading to a correctly folded (and stable) unit ready for oligomerization.

Next, to explore MucR oligomeric assembly, we used the AlphaFold2-Multimer algorithm (AF2) (47), and produced a model for a 12-protomers NTD oligomer, which is the predominant oligomerization form identified in our cryo-EM data. The resulting 12-mer assembly resembles a hollow truncated cone with diameters of 24 and 48 Å for the two bases, respectively (Figure 2C). α1 of each protomer lies almost parallel to the truncated cone axis and faces the inner face of the oligomer; α2 points mostly towards the outer face. The whole assembly is held together mainly by hydrophobic interactions: the α1 helix of each protomer exposes a continuous hydrophobic patch that interacts with residues placed on the opposite surface of α1 of the following protomer; similarly, α2 faces hydrophobic residues of the next protomer (Supplementary Figure SI5). The lower rim and a substantial part of the lower and inner open surfaces are negatively charged (Figure 2D). The 12-mer AF2 model is fully compatible, in size and shape, with the single particle cryo-EM 2D averages described above, and suggests that MucR oligomerization does not require the C-terminal DBD, with the NTD being sufficient to assemble the full multimeric unit.

**Figure 3.**
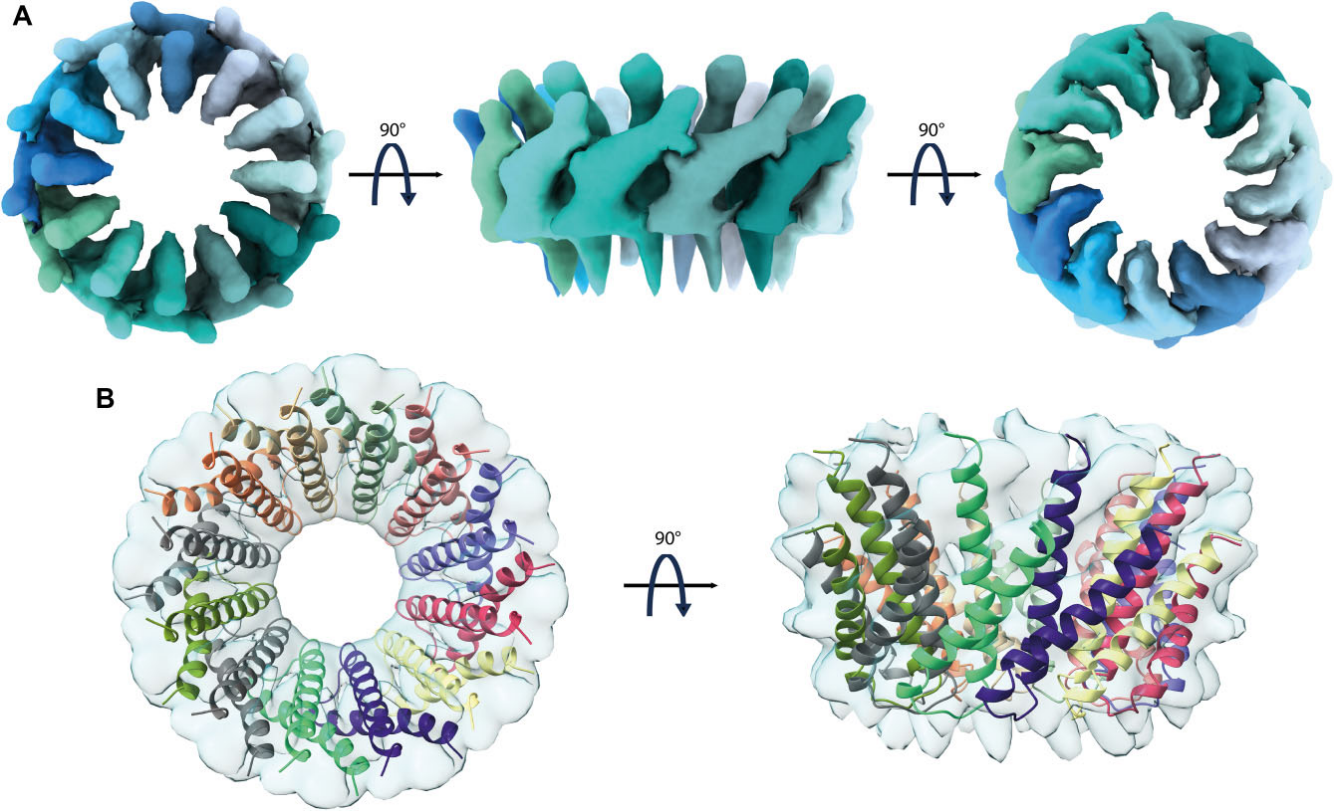
(**A**)**MucR NTD**. MucR NTD cryo-EM reconstruction at 8 Å resolution in two orientations. (**B**)**MucR NTD**. AF2 model of the MucR NTD dodecameric complex fitted onto the cryo-EM density map.

We then selected among the different MucR cryo-EM 2D classes those representative of a 12-protomer closed-circle structure (179 035 particles) and generated an *ab initio* 3D reconstruction to avoid model bias, applying a soft mask local refinement to minimize overfitting (Supplementary Figure S1A). The initial reconstruction showed an interpretable continuous low-resolution signal for the two α-helices and the connecting linker (Supplementary Figure S1B). Nevertheless, the final reconstructed volume suffered from anisotropy and some overfitting due to preferential orientation issues (Supplementary Figure S1B). Hence, to avoid over-interpretation, we low-pass filtered the final reconstruction to 8 Å (Figure 3A). Finally, we superposed the predicted AF2-multimer MucR dodecameric assembly on the cryo-EM reconstructed volume (Figure 3B). When the original AF2 model was fitted into the cryo-EM density, the best correlation coefficient (0.701) was obtained for a tilt angle of ∼39° between α1 and α2 helices, about 6° lower than the value obtained for the AF2-multimer model. This observation suggests that the two helices may adjust their relative juxtaposition, in keeping with the cryo-EM 2D images that show some heterogeneity in MucR oligomeric assembly. It also suggests that the presence of the DBD (albeit not visible in the cryo-EM particles) might have some impact on the structure of the NTD protomers upon quaternary assembly (see below).

**Figure 4.**
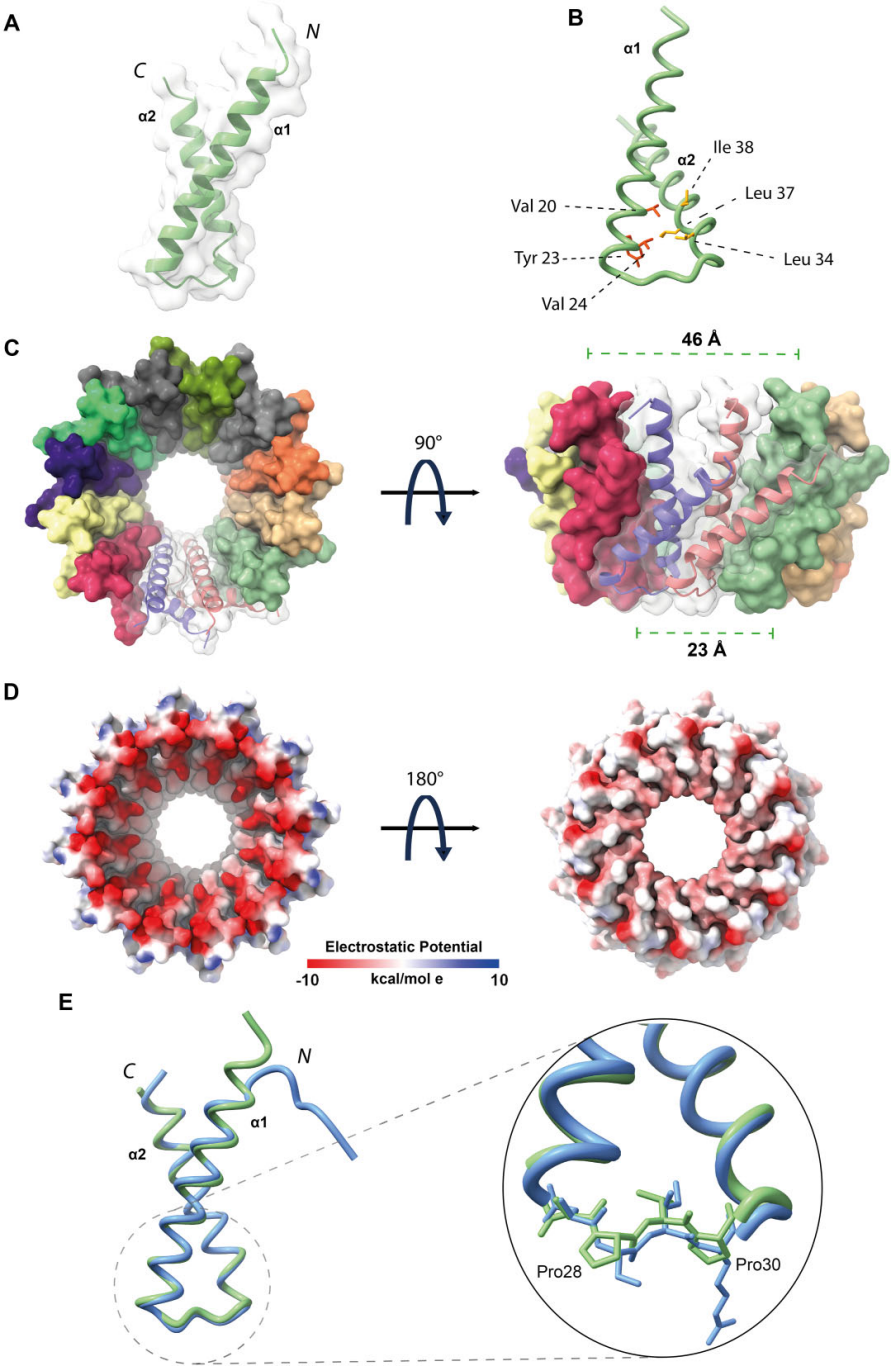
(**A**)**Ml5 NTD**. AF2 model of the Ml5 NTD (Ml5_1-56_). (**B**)**Hydrophobic interactions**. Hydrophobic residues stabilizing Ml5 NTD helices packing. Sidechains of Leu34, Leu37 and Ile38, crucial for NTD oligomerization, are depicted in orange, while sidechains of Val20, Tyr23 and Val24, necessary for the hydrophobic packing are in red. (**C**)**Ml5 NTD complex**. Surface representation of AF2 model of the Ml5 NTD dodecameric complex. Each protomer is depicted with a different color and the secondary structure elements of two protomers are presented as ribbons. (**D**) Top and bottom view of the electrostatic potential surface of Ml5 NTD dodecameric complex. (**E**)**MucR and Ml5 NTDs**. Overlay of MucR_1-56_ (in blue) and Ml5_1-56_ (in green) AF2 models. The inset shows a closer view of the loop connecting the two helices for both models. The sidechains of the amino acid residues are shown as sticks, Pro28 and Pro30 of Ml5 are labeled.

Next, AF2 was also used to generate a model of the protomeric Ml5 NTD, which, as expected, hosts two helices: α1 spans residues Glu3-Asn26, and α2 residues Val31-Gly51 (Figure 4A and B). The Ml5 model displays a helical tilt of ∼44°. We then generated an AF2-Multimer model of dodecameric Ml5 NTD (Figure 4C), which appeared highly similar to the MucR hollow truncated cone structure (with diameters at the rims of 46 and 23 Å, respectively), the wider rim being less negatively charged (Figure 4D). In the Ml5 model, α1 is connected to α2 through a loop built by Asn27, Pro28, Val29 and Pro30; the same loop instead hosts Asn29, Ser30, Ile31, Arg32 in MucR (Figures 1B and 4E). The presence of two Pro residues suggests lower flexibility for the Ml5 loop compared to that of MucR. Normal mode analysis run on MucR and Ml5 NTD models is in keeping with such a hypothesis, as the interhelical α1–α2 loop in MucR presents a higher degree of conformational flexibility, with a per-residue Root Mean Square Fluctuation value RMSF_avg29–32_= 3.21 ± 0.17 Å versus Ml5 RMSF_avg27–30_= 2.85 ± 0.4 Å (Supplementary Figure S6). The restricted mobility of the loop connecting the two helices in Ml5 might constrain the relative position of the two helices in an orientation that allows preferential formation of circular particles of 12 promoters compared to the wider palette of MucR assembly.

### Structural characterization of MucR and Ml5 DBDs

The conformational heterogeneity explored by the linker connecting NTD and DBD did not allow cryo-EM visualization of the DBD of MucR and Ml5 in the context of the full-length proteins (Figure 1A and D). For this reason, structural characterization of the isolated DBDs was performed by both CD and NMR spectroscopy, and coupled with AF2 predictions. We generated the DBD constructs MucR_57–142_ and Ml5_56–154_ based on the homologous *A. tumefaciens* Ros_56–142_ (Figure 1B), whose structure had already been solved by NMR (PDB-code 2JSP) (41).

**Figure 5.**
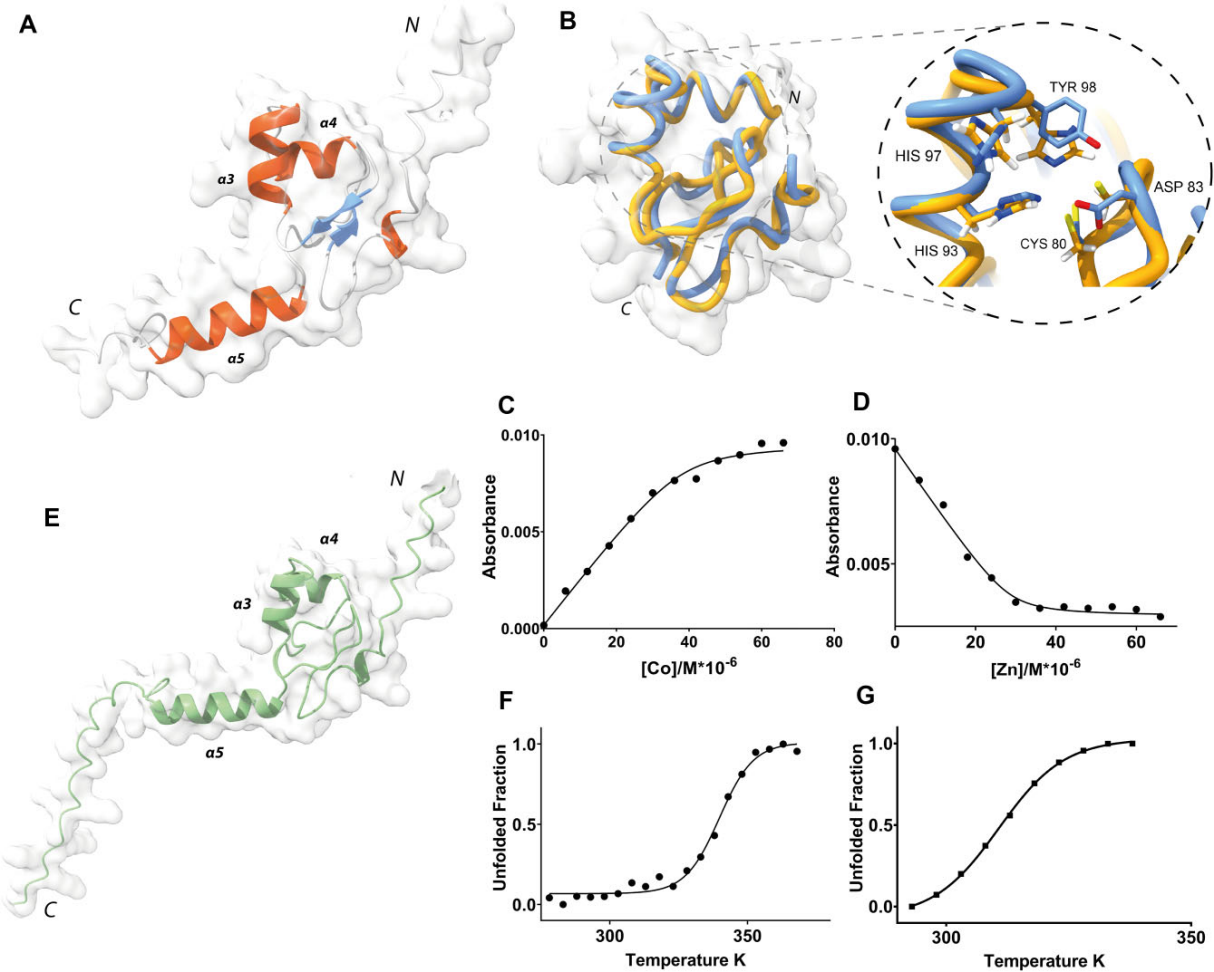
(**A**)**MucR DBD**. Ribbon drawing of the AF model of the MucR DBD. The secondary structure elements are depicted in red (α-helices) and blue (β-strands). (**B**)**MucRand Ros DBDs**. Superposition of the globular domains of Ros_56–142_ (orange) and MucR_57–142_ (blue). The inset shows a closer view of the zinc coordination sphere. Sidechains of Cys80, Asp83, His93, His97 and Tyr98 of MucR (and the corresponding residues of Ros (see Figure 1B) are depicted as sticks. (**C**)**Co(II) binding**. Titration of MucR_57–142_ with CoCl_2_ monitored at 352 nm. Absorbance is plotted against cobalt concentration. Data have been fitted using a binding isotherm as reported in the ‘Materials and methods’ section. (**D**)**Zn(II) binding**. Reverse titration of Co(II)-MucR_57–142_ with ZnCl_2_. Absorbance is plotted against zinc concentration. Data have been fitted using a binding isotherm as reported in the ‘Materials and methods’ section. (**E**)**Ml5 DBD**. Ribbon drawing of the AF2 model of the Ml5 DBD. (**F**)**Thermal unfolding of MucR_57–142_**. Melting curve of MucR_57–142_ monitored by changes in CD ellipticity at 222 nm. (**G**)**Thermal unfolding of Ml5 DBD**. Melting curve of Ml5 DBD monitored by changes in CD ellipticity at 222 nm.

The MucR_57–142_ [^15^N,^1^H] HSQC spectrum showed good chemical shift dispersion in both nitrogen and proton dimensions (Supplementary Figure S7A), confirming a well-defined native structure in solution that allowed for a nearly complete chemical shift backbone assignment (HN, N, Cα and Cβ). Chemical shift deviation (CSD) analysis (Supplementary Figure S7B and Supplementary Table S3) confirms that MucR_57–142_ contains a βββαα motif. Interestingly, the Asn121–Met133 region (α5), which includes basic residues shown to be important for the DNA binding, has a clear α-helical propensity (42,56). MucR_57–142_ structural model computationally predicted by AF2 (Figure 5A) has been compared with this NMR analysis. The Cα and Cβ Q-factor values (see the ‘Materials and methods’ section and Supplementary Figure S7C), indicate that the obtained AF2 model is in good agreement with the NMR experimental data. The average value of 9.98 ± 0.8 (s^−1^) for the assessed R_2_ obtained by analyzing the ^15^N linewidths in the [^1^H-^15^N] HSQC spectrum agrees well with that estimated by the AF2 model [10.16 ± 1.5 (s^−1^)], further indicating that it properly describes MucR_57–142_ behaviour in solution: a globular folded domain flanked by flexible N- and C-terminal tails. In particular, the MucR_57–142_ globular region consists of 55 amino acids (residues 65–119) stabilized by a large 14-residue hydrophobic core and flanked on both sides by two flexible tails. In good agreement with the cryo-EM data, the MucR_57–142_ N-terminal tail, corresponding to the linker connecting DBD and NTD, is poorly structured. The C-terminal tail, in accord with the CSD analysis, harbors a helical region encompassing residues Asn121-Met133. Interestingly, the zinc-binding domain of MucR_57–142_, which in Ros_56–142_ tetrahedrally coordinates Zn(II) through the typical Cys_2_His_2_ coordination, is formed by a CysAspHis_2_ coordination sphere (Figure 5B) (42,68). MucR_57–142_ characterization provides the first structural description of a native protein exploiting the metal binding properties of an aspartate within a CysAspHis_2_ coordination sphere. To investigate its binding affinity to the metal ion in MucR_57–142_, which is mostly unstructured when the ion is absent (Supplementary Figure S8), we have estimated the zinc dissociation constant (*K*_d_*) (Figure 5C and D) obtaining values of 1.34 (±0.40) x10^−6^ M and 1.20 (±0.50) x10^−8^ M for Co(II) and Zn(II), respectively. These values are comparable to *K*_d_* observed for Ros_56–142_ despite the different Zn(II) coordination sphere (69).

To analyze the effect of the CysAspHis_2_ coordination sphere on the protein stability, a thermal unfolding characterization of MucR_57–142_ was carried out. Overall, the behavior observed is indicative of a two-state thermal unfolding with a melting temperature of 339.8 (± 0.8) K (Figure 5F) consistent with a folding/unfolding conformation exchange on the micro-millisecond timescale (68,70,71). Ros_56-142_ unfolding has been shown to be a different, two-step process in which a metal binding intermediate converts to the native state through a downhill barrier-less transition (69). Therefore, our data suggest that the different zinc coordination sphere implies a different thermodynamic behaviour of MucR_57–142_ with respect to Ros_56–142_.

The Ml5_56–154_ [^15^N,^1^H] HSQC spectrum, by analogy with MucR_57–142_, showed good chemical shift dispersion in both nuclei dimensions (Supplementary Figure S9), again confirming a well-defined native structure in aqueous solution for the DBD domain. Thus, AF2 was used to obtain a model of Ml5_56–154_ structure (Figure 5E). We have estimated also in this case the R_2_ by analyzing the [^1^H-^15^N] HSQC ^15^N linewidths obtaining the average value of 10.93 ± 1.4 s^−1^ that is in agreement with the value estimated by the obtained model (10.33 ± 1.8 s^−1^), indicating that also in this case it properly describes the behaviour of Ml5_56–154_ in solution. Next, also for Ml5_56–154_ the thermal unfolding characteristics were investigated. CD and NMR data clearly reconcile in demonstrating also for this zinc-free protein a two-state thermal unfolding behaviour (Figure 5G) with a melting temperature of 310.7 (± 0.3) K thus showing that the zinc-free Ml5_56–154_ is less thermo-stable than the zinc-bound MucR_57–142_. Ml5_56–154_ shows a globular domain with an overall structure similar to that of Ros_56–142_ and MucR_57–142_ with minor local rearrangements necessary to preserve the global fold. Like these latter two proteins, Ml5_56–154_ is flanked by N- and C-terminal flexible tails. A set of polar and hydrophobic interactions, involving residues located within the spatial region where the zinc is bound in Ros_56–142_ and MucR_57–142_, stabilize the Ml5_56–154_ fold in the absence of a zinc-binding site, in agreement with previous mutagenesis experiments (72).

**Figure 6.**
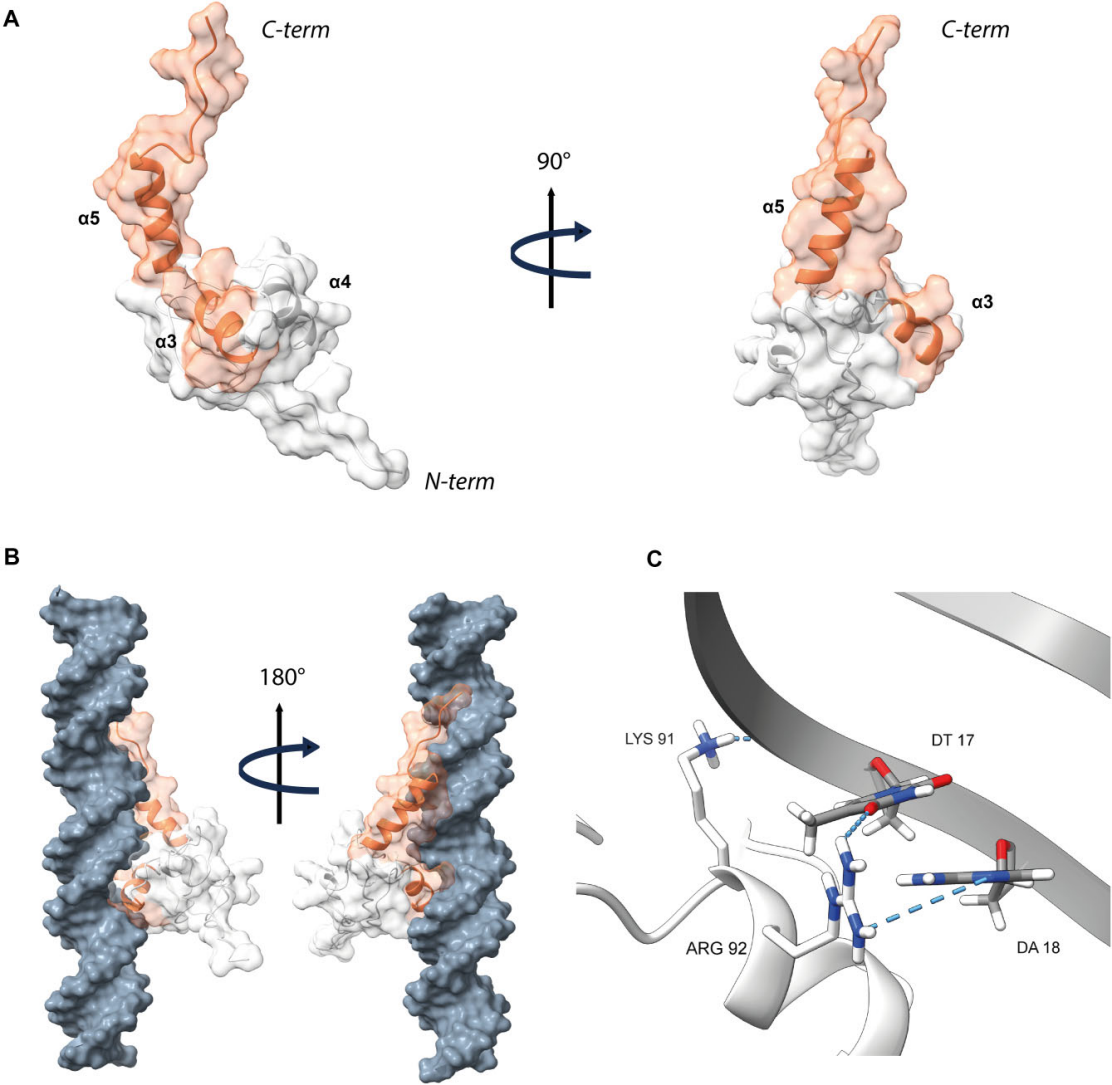
**MucR–DNA complex**.(**A**) Residues of MucR_57–142_ that interact with DNA mapped (in red) on the surface of the AF2 model. (**B**) Surface representation of a typical HADDOCK model for the MucR_57–142_–DNA complex. The protein interaction surface is depicted in orange. (**C**) Close-up view of the main interactions stabilizing the MucR_57–142_–DNA complex. Side-chains of Lys91 and Arg92 of MucR_57–142_ and nucleobases of T17 and A18 of the DNA are shown as stick.

To obtain structural insights into complexes of the DBD of Ros/MucR family members with DNA, we first investigated *in silico* the interaction of MucR_57–142_ with a 30 bp AT-rich oligonucleotide (babR30) derived from an identified target site in the *babR* gene promoter (28) (Supplementary Figure S10). The residues predicted to build the protein/DNA interface are in agreement with the binding regions previously identified by mutagenesis experiments and *in silico* studies on MucR homologs (42,56,73). The MucR_57–142_/DNA binding interface (Figure 6A) is mainly composed of residues in α3, which insert into the major groove providing contacts with DNA bases and backbone phosphates (Figure 6B). In particular, the side chain of residue Lys91 is within hydrogen bonding distance of oxygen atoms of the phosphate backbone (Figure 6C), whereas the side chain of Arg92 appears to be involved in base interactions with bases T17 and A18 (Figure 6C). MucR_57–142_ further contacts the DNA, interacting with the minor groove through the C-terminal α5 containing Arg126 and Arg128, which constitute a conserved basic region demonstrated to be important for DNA binding of MucR homologs (42,73) (Figure 1B). These data are in line with previous results demonstrating that the Ros/MucR family members bind AT-rich sites containing TpA steps within their DNA targets and that the interaction with the DNA minor groove is essential for this activity (24,27,28). Moreover, the model suggests that the binding is further stabilized by residues at the end of the C-terminal tail contacting the major groove.

Overall, our model outlines a DNA binding mechanism where MucR_57–142_ interacts with a 16 bp extended recognition DNA site within a 30 bp double-stranded oligonucleotide. This mechanism of DNA binding can be extended among MucR homologs in light of their high degree of sequence identity (Supplementary Figure S4).

### Full length MucR/Ros oligomers assemble through a central scaffold core decorated with DBDs

**Figure 7.**
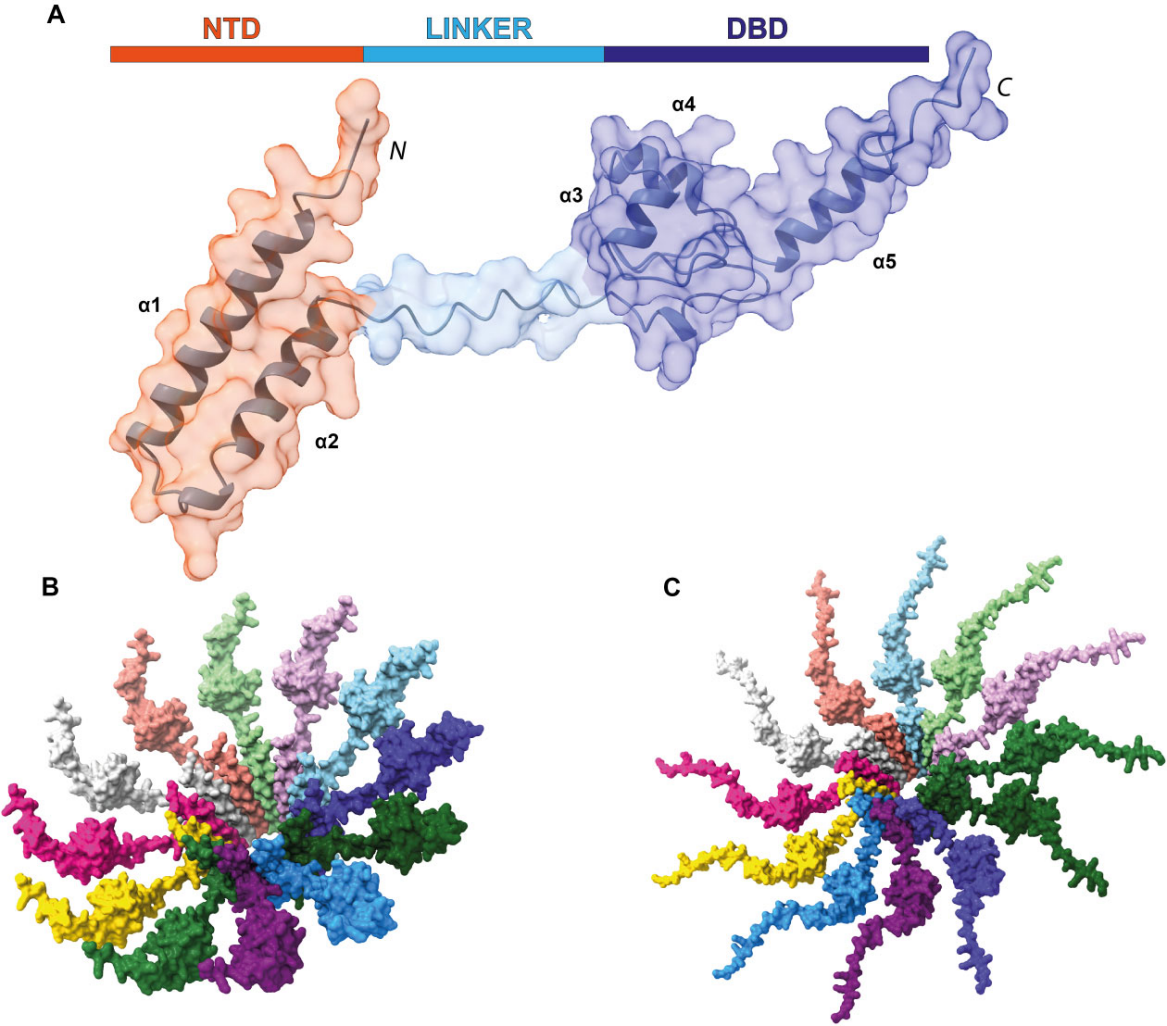
(**A**)**MucR full-length**. Surface representation of AF2 model obtained for the full-length monomer of MucR. The two independent domains and the flexible linker are highlighted and shown in different color. (**B**)**MucR and**(**C**)**Ml5 particles**. A model for the MucR and Ml5 full dodecameric particles. Each monomer is depicted with a different color.

To further our understanding of the assembly and functional implications of MucR and Ml5 as prototypes of the MucR/Ros family, we started from the structural model of a monomeric unit of MucR. We predicted the 3D model of the full-length monomeric MucR (NTD + DBD) using AF2 (Figure 7A). The obtained model shows a bi-lobal conformation in which the DBD is connected through a flexible poorly structured region (Figure 7A and Supplementary Figure S11) to the NTD that folds in two antiparallel helices. This model, in agreement with the above reported experimental data, explains the conformational heterogeneity explored by DBDs in the cryo-EM analysis.

The NTD structure within the full-length monomeric protein obtained by AF2 is superimposable (RMSD_3-52_= 0.7 Å) with the NTD present in the model of the 12 NTD oligomeric structure based on cryo-EM results (Supplementary Figure S12). Thus, the latter was used as a template for the assembly of a model for the oligomeric structure of full-length MucR (Figure 7B): the NTD regions of 12 full-length monomeric proteins were overlaid, by means of ChimeraX, with the same region of each protomer in the 12 NTD oligomeric structure.

The obtained model shows a central hollow truncated cone in which the zinc-binding DBDs decorate the external part protruding from the wider rim of the assembly suggesting that the DBDs could freely move and interact with the surrounding DNA.

Very similar results were obtained when the modeling exercise was run on full-length Ml5 (Figure 7C), suggesting that the assembly architecture here presented for MucR and Ml5 may have a general validity for the proteins of the MucR/Ros family.

### MucR and Ml5 oligomers bridge AT-rich DNA

**Figure 8.**
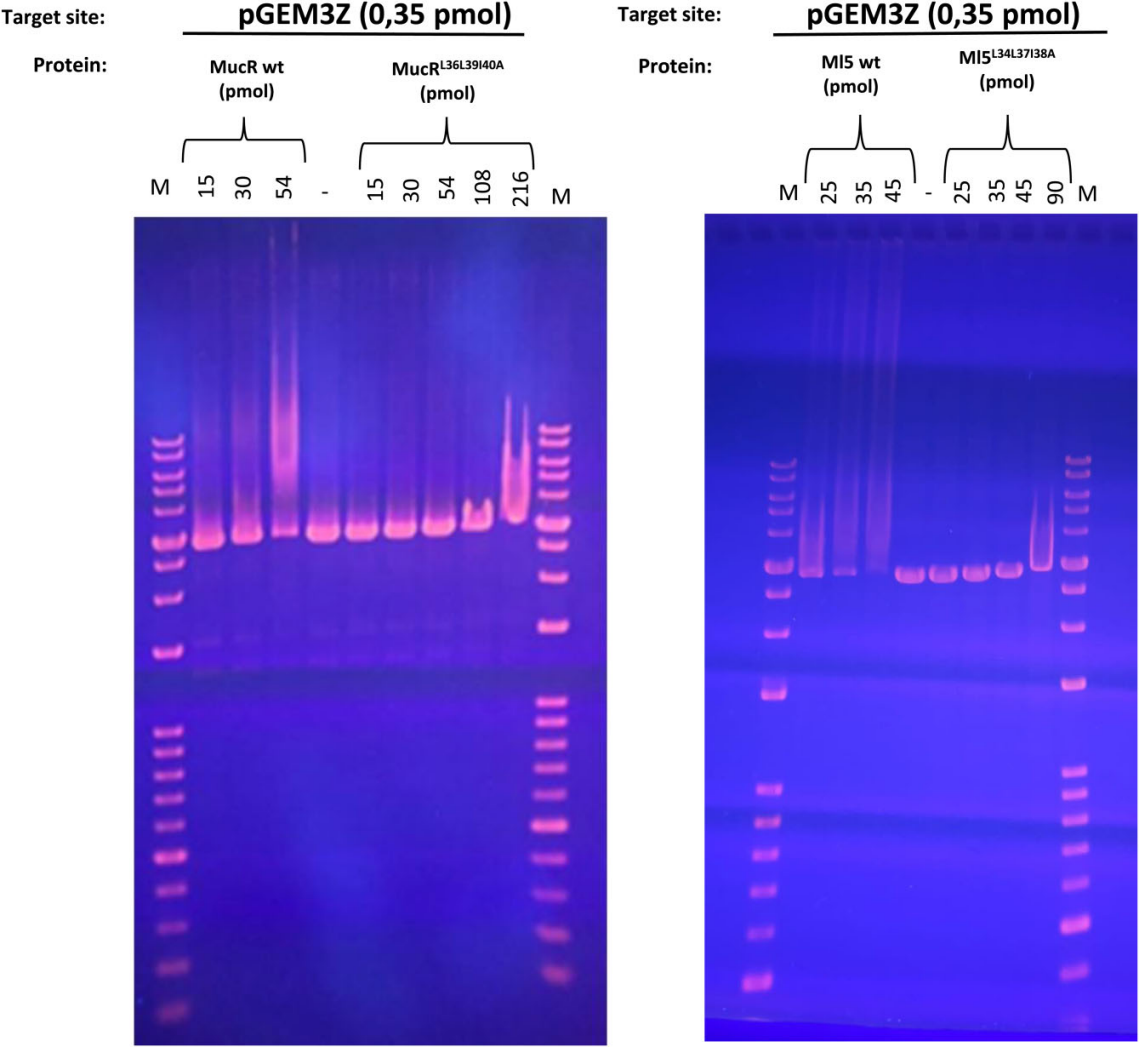
EMSA of MucR, MucRL36L39I40A (left), Ml5 and Ml5L34L37I38A (right) using the linearized pGEM-3Z as a target. At the top of the lanes, M indicates the 1 kb DNA marker (Bioline); numbers indicate pmol of protein used in the binding reaction. MucR clearly binds the pGEM3Z target when 154:1 protein:DNA ratio is used (54 pmol of protein and 0.35 pmol of DNA were used for the binding reaction), whereas its mutant version can bind only when reaching a 617:1 protein:DNA ratio (216 pmol of protein and 0.35 pmol of DNA were used for the binding reaction). Ml5 binds pGEM-3Z at 74:1 protein:DNA ratio and the target is totally bound when 134:1 protein:DNA ratio is used (26 pmol of the protein and 0.35 pmol of DNA were sufficient to detect binding; using 47 pmol of protein, the DNA is totally bound). Ml5^L34L37I38A^ binds pGEM-3Z only when 257:1 protein:DNA ratio is used (90 pmol of protein and 0.35 pmol of DNA were used for the binding reaction). pmol of MucR and Ml5 were calculated on the base of the MW of the proteins calculated by LS analysis in Pirone *et al.* 2018 (26) and this study, respectively; pmol of MucR^L36L39I40A^ and Ml^5L34L37I38A^ were calculated considering the MW of the monomers, these variants being unable to form oligomers.

To shed light on the functional role played by Ros/MucR oligomerization assembly we challenged the DNA-binding properties of wild-type MucR and Ml5 *versus* their variant impaired for oligomerization (MucR^L36L39I40A^ and Ml5^L34L37I38A^). Considering the positive effect of DNA length on MucR binding (65), first we used EMSA with a long DNA target, the pGEM-3Z linearized plasmid, which is 50% AT-rich (Figure 8). The results show that at the same DNA concentration, lower concentrations of the wild-type proteins are required to observe DNA binding, compared to that of the mutants. Similar results were obtained when EMSAs were performed using a shorter dsDNA oligonucleotide, the 60 bp AT-rich babR60, previously shown to be a MucR target site in the *babR* gene promoter (28). Altogether, these results demonstrate that MucR and Ml5 oligomerization is necessary for higher affinity DNA binding (Supplementary Figure S13). Furthermore, we show that MucR and Ml5 share a clear preference to bind AT-rich DNA targets, by comparing in EMSAs their DNA binding to babR30, a 30 bp 83% AT-rich oligonucleotide derived from babR60, and NS, a 30 bp scrambled GC-rich oligonucleotide (20% AT content) (Supplementary Figure S10).

**Figure 9.**
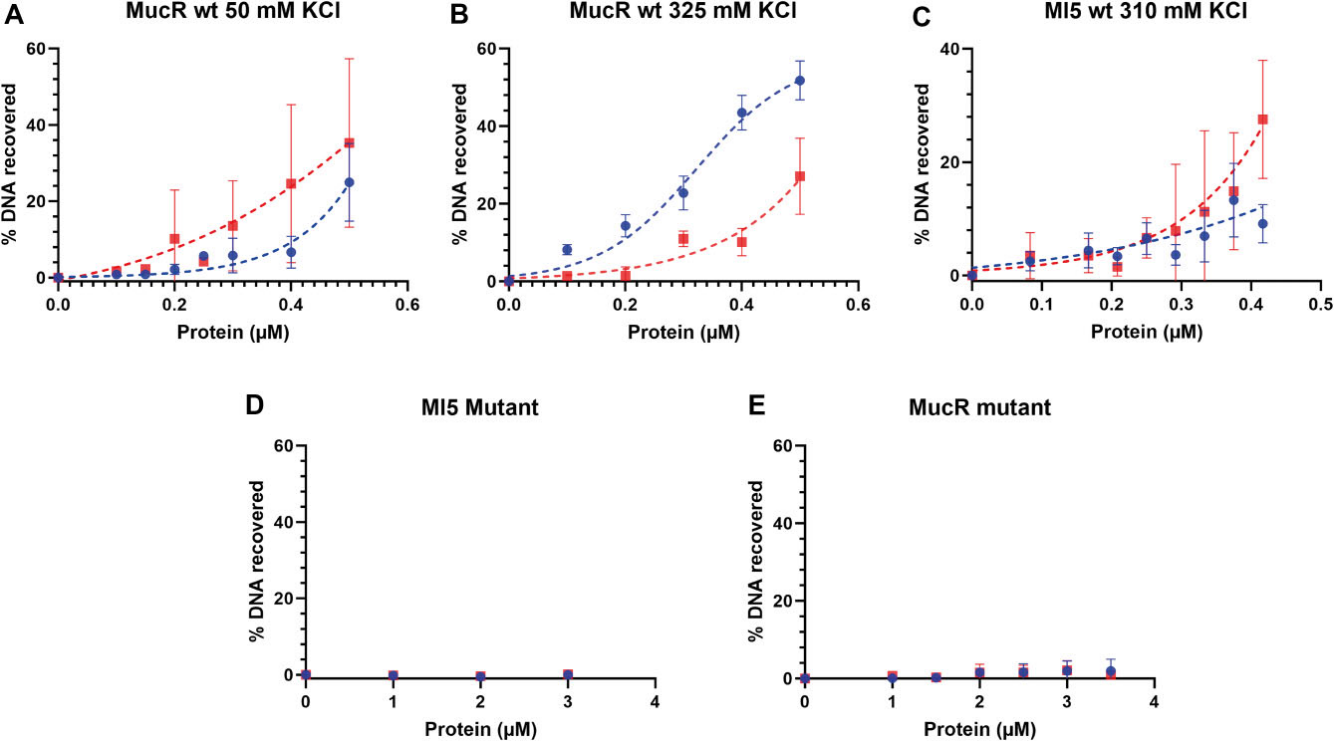
**DNA-bridging efficiency as function of protein concentration using two 685 bp DNA probes**. Within the same GC-rich sequence, one probe (represented by blue circles) contains the *mucR* promoter sequence, while the other probe (represented by red squares) contains the babR60 sequence (Supplementary Table S1). Dashed lines are to guide the eye only and are not a representation of an underlying molecular model. Concentrations were determined based on oligomeric state as determined by LS analysis shown in Pirone *et al.* (26) for MucR and in this study for Ml5. (**A**) MucR wt was titrated into an experimental buffer with a final concentration of 50 mM KCl. (**B**) MucR wt was titrated into an experimental buffer with a final concentration of 325 mM KCl. (**C**) Ml5 was titrated into the assay with an experimental buffer containing 310 mM KCl. (**D**) MucR^L36L39I40A^ did not show any DNA recovery in 50 mM KCl. (**E**) Likewise, Ml5^L34L37I38A^ did not yield detectable DNA–protein–DNA interactions.

Next, to demonstrate that MucR is able to bridge two separate DNA duplexes, we performed a DNA bridging assay with wild-type MucR and the mutant MucR^L36L39I40A^ variant using the *mucR* gene promoter, which is a demonstrated MucR target (25,33), and the babR60 sequence (Figure 9). The two targets were cloned into pBHT154 (63). The resulting vectors were used to generate the two 685 bp substrates for bridging assays by PCR. Our experiments show that MucR bridges DNA efficiently (Figure 9A), and bridging is strictly dependent on whether the protein can oligomerize (Figure 9D); these results on *B. abortus* MucR are in agreement with those published for *S. fredii* MucR (27).

We noticed that the error bars for the experiments with 50 mM KCl reported in Figure 9A are quite large. With our control samples we observe non-specific interactions between the DNA–protein complexes and the beads, which is an indication for either aggregation or precipitation of proteins. To decrease the observed measurements errors, we increased the KCl concentration to 325 mM (Figure 9B), yielding smaller error bars. Interestingly, DNA recovery for the babR60 probe remains unaltered, while we observe an increase for the *mucR* promoter. The difference between the two substrates may be attributed to small differences in geometry/structure of the complexes due to differences in DNA sequences. Overall, our data indicate that MucR is able to bridge two DNA duplexes in solution.

Bridging assays were also performed for Ml5 in a buffer containing 310 mM KCl, which helped to avoid non-specific interactions with beads. Similar to MucR, Ml5 bridges DNA (Figure 9C). Bridging experiments with MucR^L36L39I40A^ and Ml5^L34L37I38A^ demonstrate that these variants are much less proficient in DNA–protein–DNA interactions compared to the wild-type proteins (Figure 9D and E). These results are in line with those obtained by EMSAs and underline the strict dependence of DNA bridging on oligomerization.

### MucR and Ml5 oligomers compact DNA

**Figure 10.**
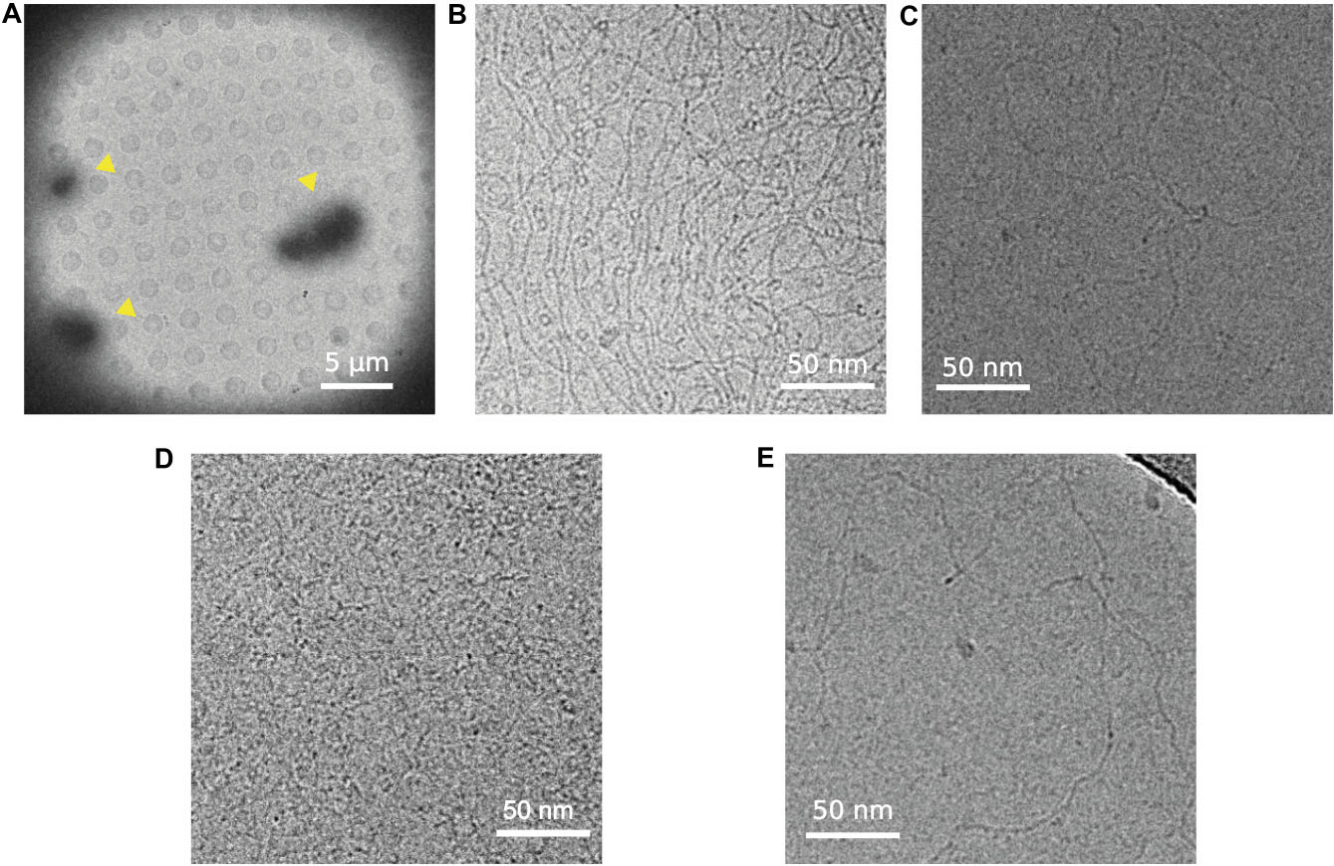
**MucR–DNA and Ml5–DNA complexes**.(**A**) MucR-pGEM-3Z image at low magnification (700 ×). Three clusters are indicated with yellow arrows. (**B**) MucR-pGEM-3Z plasmid complex image at high magnification (73000 ×). MucR particles can be identified as 6 nm circular particles and the DNA as fibers. (**C**) MucR^L36L39I40A^ with pGEM-3Z plasmid image at high magnification (73000 ×). No circular particles are visible, and DNA is visible as separated filaments. **(d)** Ml5-pGEM-3Z plasmid complex image at high magnification (73000 ×). Ml5 particles can be identified as 6 nm circular particles. (**E**) pGEM-3Z plasmid image at high magnification (73000 ×).

To obtain further insights into the DNA binding properties of MucR, we analyzed the protein–DNA complexes through cryo-EM experiments (Figure 10). We mixed MucR and the linearized pGEM-3Z plasmid in a 45:1 molar oligomeric protein/DNA ratio. Images of the vitrified sample at low magnification clearly show the formation of micrometer sized clusters likely due to binding of MucR to DNA (1–20 μm) (Figure 10A). Indeed, a closer inspection at the edges of such clusters revealed co-localization of particles with characteristics of MucR oligomers (6 nm circular particles) and DNA molecules (as fibers) (Figure 10B). In fact, as one single packed DNA molecule of linearized pGEM-3Z plasmid cannot generate such a large object, the results suggest that MucR has the capacity to bind and pack together multiple individual DNA duplexes through a bridging mechanism. The same experiment on MucR^L36L39I40A^ demonstrated that MucR–DNA clusters are not formed with this oligomeric deficient variant. Indeed, isolated DNA filaments could be clearly distinguished in extended conformation and micrometer-sized clusters are absent (Figure 10C). We further investigated the MucR DNA-clustering capacity with shorter DNA sequences, the 232 bp *mucR* promoter and babR60. In presence of both (AT-rich) shorter oligonucleotides, MucR was able to form micrometer-sized protein–DNA clusters (Supplementary Figure S14). Remarkably, besides the clusters, no isolated DNA molecules or MucR particles could be detected, highlighting strong capacity of MucR to bind DNA.

We then analyzed the interaction between Ml5 and the linearized pGEM-3Z plasmid in a 45:1 oligomeric protein/DNA ratio, vitrified the sample and imaged on a TEM at cryogenic temperature. As for MucR, Ml5–DNA formed micrometer-sized clusters (Figure 10D) and no isolated Ml5 or DNA molecules were visible.

In conclusion, we were able to visualize the DNA bridging and compacting capacity of the Ros/MucR family members. The results clearly show that these activities are strictly dependent on Ros/MucR proteins oligomerization.

## Discussion

This study reports on the extensive structural characterization of the H-NS-like protein MucR from *B. abortus* and its homolog Ml5 from *M. loti* using different experimental techniques and *in silico* analyses. In addition, we investigate the ability of both proteins to bridge DNA uncovering the importance of their quaternary structure in this function. The two proteins form closed-circular and horseshoe-shaped oligomers in which the N-terminal oligomerization domains build a hollow truncated cone hub with the C-terminal DBDs protruding from the cone wider rim. The two independent domains are connected by a flexible linker that allows the DBDs to freely move and bind DNA in the surrounding space. The structural characterization of the zinc-binding MucR and of the zinc-free Ml5 DBDs shows that, although the two domains share the same two state unfolding mechanism and the same ability to bind and bridge DNA, the presence of a structural zinc ion confers a higher thermal stability to MucR. As most of the Ros/MucR proteins have DBDs similar to the one present in *B. abortus* MucR, the higher stability of the zinc-binding domains might reflect a positive evolutionary selection.

**Figure 11.**
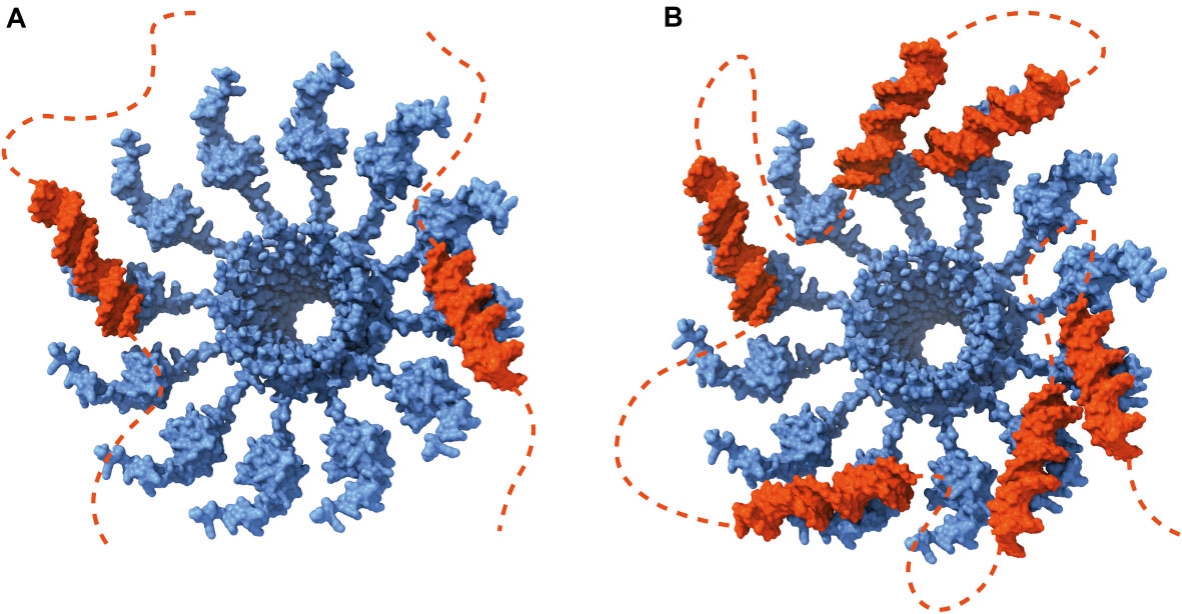
**Proposed MucR–DNA bridging mechanism**. Cartoon representation of MucR–DNA *in trans***(a)** and *in cis***(b)** bridging interactions, respectively.

The oligomerization of MucR and Ml5 forms a quaternary structure where the DBDs from multiple monomers participate in both DNA binding and bridging. The circular structures formed by MucR and Ml5 might function as molecular platforms that allow their DBDs to simultaneously interact with two different DNA duplexes mediating *in trans* interactions (Figure 11A). Furthermore, this structure also suggests the possibility that MucR and Ml5 can mediate *in cis* bridging interactions (Figure 11B). Indeed, AT-rich sequences may work as multiple nucleation sites and, when present on the same double-stranded DNA, would allow DNA to wrap around the protein oligomers. This elegant and simple way to bridge DNA could explain how these proteins organize the structure of nucleoids and hinder the access to the transcriptional machinery at gene promoters or trap the RNA polymerase, as reported for H-NS-like proteins (1,2,15). The ability to bridge DNA also is compatible with other models of transcription repression during transcription elongation relying on topological stress (74,75).

The high level of sequence identity between members of the Ros/MucR family and their ability to oligomerize, bind and bridge AT-rich DNA lead us to re-consider the role of this family as a distinct unique sub-family of H-NS-like proteins. Classic H-NS and H-NS like proteins, mostly identified in β- and γ-proteobacteria, form long protein filaments along the bacterial genome, and the presence of multiple DBDs in these filaments allows to bridge and stiffen DNA. The circular structure of Ros/MucR proteins provides them with the same functionality in terms of DNA organization, albeit with a different mechanism that relies on a peculiar circular oligomeric protein assembly. Our results confirm the differences of oligomeric structures between Ros/MucR proteins and the classical H-NS described by Shi *et al.* (27), but also extend our knowledge on the ability of Ros/MucR family members to form closed-circular oligomers, which support DNA bridging and compaction. Basic residues located on the C-terminal tail of Ros/MucR proteins, are fundamental for DNA binding and they are responsible for interactions with the minor groove (42,73), as also supported by the MucR/DNA docking complex. This is a shared feature with other H-NS and H-NS-like proteins, which establish pivotal contacts with the minor groove through an arginine residue located in their DBDs (5,76). Nevertheless, unlike classical H-NS and H-NS-like proteins, Ros/MucR proteins also contact the major groove of DNA underlying another difference in their DNA-binding mechanism. In light of our data, the role played in gene expression regulation by the Ros/MucR proteins might be related to their ability to structure the bacterial genome rather than to act as classical transcriptional regulators.

Our findings pave the way to future studies focused on deciphering DNA structural changes induced by the atypical circular oligomers that regulate gene transcription.

## Supplementary Material

gkae1104_Supplemental_File

## Data Availability

Cryo-EM data are available in EMDB and can be accessed with the EMDB code: EMB-19826. The data underlying this article are available in the article and in its online supplementary material.
