# Correction to ‘Circular oligomeric particles formed by Ros/MucR family members mediate DNA organization in α-proteobacteria’

**DOI:** 10.1093/nar/gkae1269

**Published:** 2024-12-19

This is a correction to: Antonio Chaves-Sanjuan, Gianluca D’Abrosca, Veronica Russo, Bert van Erp, Alessandro Del Cont-Bernard, Riccardo Capelli, Luciano Pirone, Martina Slapakova, Domenico Sgambati, Roberto Fattorusso, Carla Isernia, Luigi Russo, Ian S Barton, Roy Martin Roop, Emilia M Pedone, Martino Bolognesi, Remus T Dame, Paolo V Pedone, Marco Nardini, Gaetano Malgieri, Ilaria Baglivo, Circular oligomeric particles formed by Ros/MucR family members mediate DNA organization in α-proteobacteria, *Nucleic Acids Research*, 2024; https://doi.org/10.1093/nar/gkae1104.

In the originally published version of the manuscript, details were missing from the section **Funding**. This should read:

“Italian Research Minister [PRIN2022 P2022AW2H9 and PRIN2022 P2022K9SJ27]; National Institute of Allergy and Infectious Diseases [R01AI172822]; Netherlands Organization for Scientific Research [OCENW.GROOT.2019.012 to R.T.D.]. Funding for open access charge: Italian Research Minister [PRIN2022 P2022AW2H9 and PRIN2022 P2022K9SJ27].” instead of: “Italian Research Minister [PRIN2022 P2022AW2H9 and PRIN2022 P2022K9SJ27]; National Institute of Allergy and Infectious Diseases [R01AI172822]. Funding for open access charge: Italian Research Minister [PRIN2022 P2022AW2H9 and PRIN2022 P2022K9SJ27].].”

The emendation has been made to the article.

