# Supplementary material for: Circular oligomeric particles formed by Ros/MucR family members mediate DNA organization in α-proteobacteria
Source: Nucleic Acids Res. 2024 Nov 26;52(22):13945–63. doi: 10.1093/nar/gkae1104 (PMC11662661; doi:10.1093/nar/gkae1104)
Supplement: gkae1104_Supplemental_File [file gkae1104_supplemental_file.pdf]

Supplementary Data

**Primers used for PCR**	
PRIMER	SEQUENCE 5’-3’
1	ATCGCATATGACCGAAGAAACCGAGAGCAAAG
2	TATCGAATTCTCAGGCTGCCGCTTTCTTGCGGGC
3	CTGTGAAGCGCCTGGGGCTGCCGGCCAGATCCACATCGCATTGAAAG
4	CTGGCCGGCAGCCCCAGGCGCTTCACCGACGGGAACCGGATTGTTCGAAAC
5	GCCTCTAGACATGAGCACCGACGTCAGCAGTG
6	GTCCTGGGAGTGCCGGGAGAGCATCAGCATG
**Oligonuclotides used for Bridging Assays**	
**OLIGONUCLEOTIDE**	**SEQUENCE OF SINGLE STRAND (5’-3’)**
mucR promoter	TCTCAATTTTCTTGCGGTGCCCTGTTTAATATCATTTTATTTGTCGATCTAAG<br>AAGAGTTGCCTATTATTAATGTAATATGGTTTGACAATTCTATTGCAAATGG<br>CATCGTCAATTGATATTTCCATAAGGGATCGAGTTGGGCCGGATTATGAAA<br>TACGCAGCGGCGGCAAGGGGTGGGTTGCCATTGTCAGCCGCTGCGCGGAC<br>AACAAAAAATTAAAAAAGGAAAACTT
babR60 (stretched by 26 amino acids; see Methods)	AATTTAGAATGAAGTTATATTCAATATAAAAGTAGAATTTTGAAAATCGCTA<br>ATTATGATAATTTAGAATGAAGTTATATTCAATA

Supplementary Table 1. Primer sequences used for PCR and oligonucleotide sequences tested in bridging assays are reported.

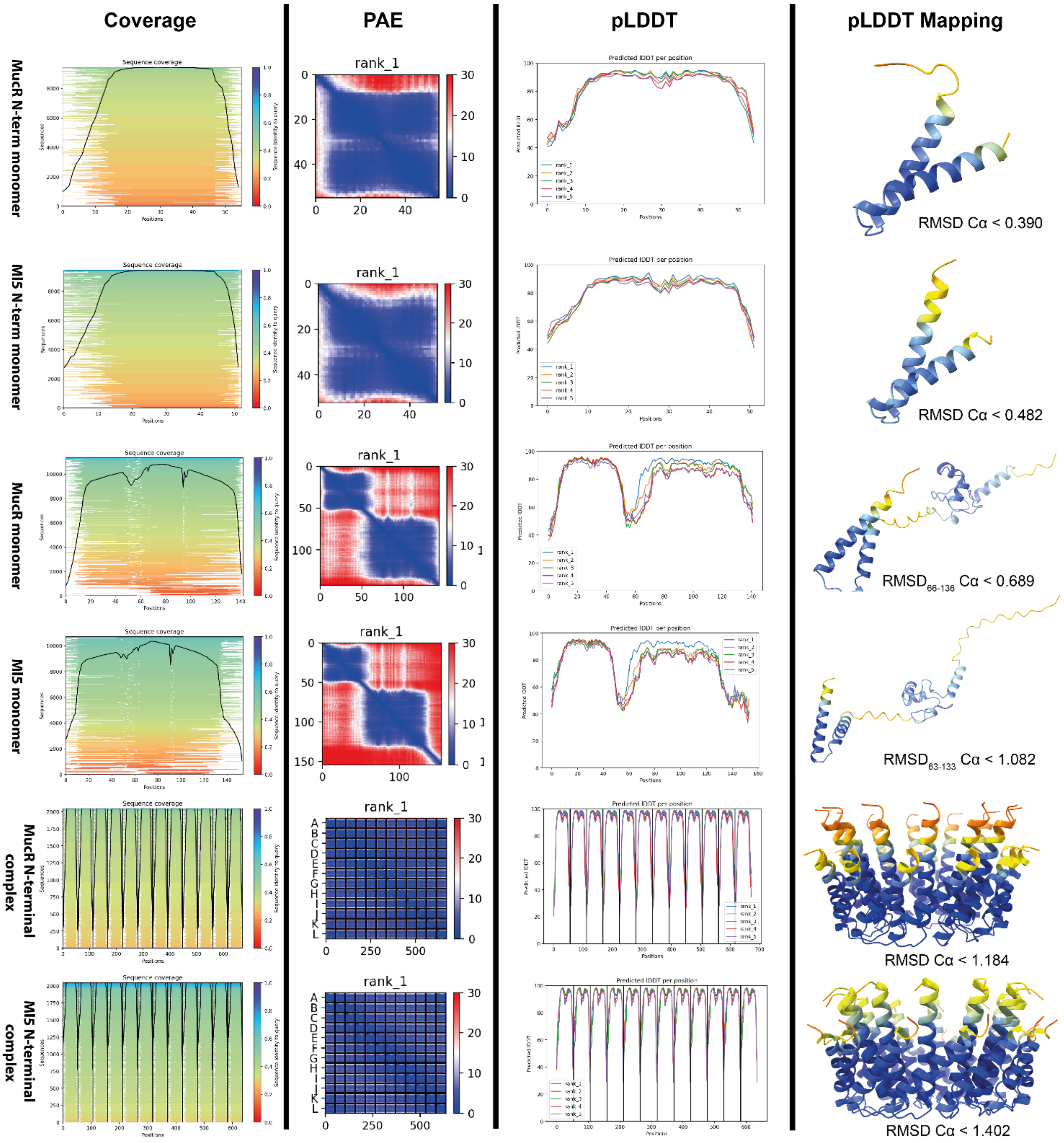

Supplementary Table 2: AlphaFold metrics. Sequence coverage, PAE map, per-residues pLDDT and pLDDT mapped on the best model are reported. Maximum Cα RMSD for the five AF2 models obtained for every query is also indicated.

Residue #	Residue type	Chemical Shift (ppm)			
		H_N	N	Cα	Cβ
58	V	-	-	60.20	30.06
59	V	8.36	125.61	59.53	30.22
60	V	8.31	125.57	59.36	30.22
61	E	8.44	125.75	53.32	28.14
62	K	8.42	124.80	51.45	-
63	P	-	-	59.92	29.42
64	K	8.65	122.96	50.73	-
65	P	-	-	59.72	29.74
66	A	8.53	121.84	52.04	17.03
67	V	7.04	106.00	55.25	33.02
68	N	8.25	119.50	48.71	-
69	P	-	-	63.07	30.06
70	K	8.43	116.94	55.35	29.42
71	K	8.23	119.33	51.80	29.82
72	S	7.62	112.60	57.12	62.35
73	V	8.27	123.36	60.84	29.58
74	H	8.65	129.33	51.32	28.86
75	D	8.11	119.08	55.54	37.18
76	D	9.03	111.90	51.41	40.45
77	Y	6.98	113.50	54.38	36.62
78	I	9.41	118.66	56.88	38.77
79	V	9.08	126.85	58.68	30.25
80	C	9.28	129.87	57.25	26.78
81	L	9.15	130.44	53.95	36.22
82	E	9.08	117.33	55.39	28.22
83	D	6.74	109.12	51.60	40.88
84	G	8.76	111.12	43.43	-
85	K	8.66	122.09	53.57	31.18
86	K	7.48	117.80	53.06	31.98
87	F	8.94	118.41	55.06	41.25
88	K	8.82	122.08	56.84	29.74
89	S	7.66	108.49	52.70	60.63
90	L	8.74	129.33	54.14	40.69
91	K	7.96	117.38	58.23	30.07
92	R	8.03	116.92	55.80	27.34
93	H	7.39	117.85	55.06	25.27
94	L	8.21	119.79	55.46	38.48
95	V	6.83	114.37	62.36	29.10
96	T	7.56	110.71	60.97	66.40
97	H	7.56	122.66	50.83	24.71
98	Y	7.71	115.57	55.97	33.18
99	N	7.99	112.39	51.79	34.86
100	M	7.16	120.43	54.58	32.06
101	T	7.98	113.60	56.50	-
102	P	-	-	63.95	30.06

103	E	8.92	115.45	58.09	26.22
104	Q	7.91	119.95	55.87	26.54
105	Y	8.56	122.83	59.64	36.70
106	R	8.62	115.88	57.41	27.26
107	E	7.80	116.74	56.31	27.34
108	K	8.15	120.60	56.31	29.66
109	W	7.15	115.03	52.85	27.02
110	D	7.59	118.01	52.69	36.62
111	L	8.62	116.96	50.25	39.65
112	D	8.60	122.53	50.44	-
113	P	-	-	62.59	29.50
114	N	8.54	112.90	49.59	35.90
115	Y	8.32	126.61	54.67	30.38
116	P	-	-	60.58	-
117	M	8.10	109.44	49.92	33.74
118	V	7.53	117.90	57.35	32.78
119	A	8.81	129.56	48.45	-
120	P	-	-	63.63	30.06
121	N	9.04	114.46	51.97	34.62
122	Y	8.04	120.39	56.40	36.22
123	A	7.94	123.29	51.07	16.31
124	A	8.13	121.28	51.00	16.23
125	A	7.94	121.76	51.06	16.23
126	R	8.13	118.49	54.48	27.66
127	S	8.10	115.78	56.84	60.88
128	R	8.12	122.11	54.73	27.90
129	L	7.96	121.30	53.14	39.57
130	A	8.03	123.45	50.47	16.39
131	K	8.06	119.56	54.15	30.30
132	K	8.17	121.60	54.03	30.06
133	M	8.32	120.46	52.94	30.22
134	G	8.33	109.43	42.76	-
135	L	8.18	121.39	52.65	39.78
136	G	8.49	109.44	42.61	-
137	R	8.09	120.28	53.08	28.30
138	K	8.45	124.37	51.55	-
139	P	-	-	60.60	29.66
140	K	8.39	120.91	54.00	30.38
141	D	8.33	121.47	51.76	38.53
142	A	7.78	128.95	51.21	-

Supplementary Table 3: NMR Chemical Shifts. Assigned chemical shifts of backbone H_N, N, Cα, and Cβ nuclei of MucR DBD.

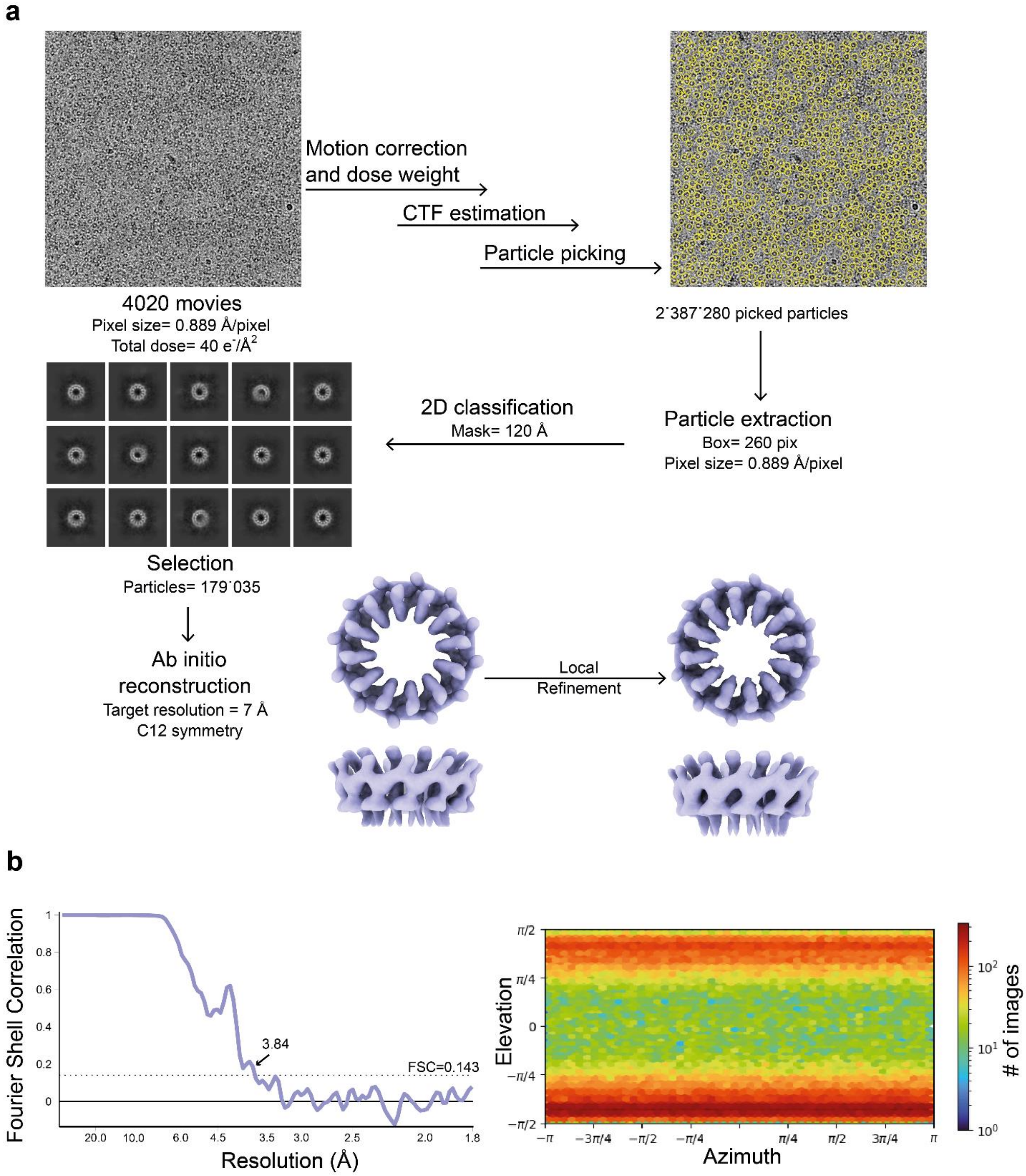

Supplementary Figure 1. Cryo-EM processing workflow. (a) The workflow is described in the methods section. Representative micrographs with and without the particle coordinates are shown. The 2D averages of the final set of particles are displayed in the selection. Two representative views of the *ab initio* reconstruction and the final reconstruction are shown. Important processing parameters are labeled below each processing step. **(b)** On the left, Fourier Shell Correlation (FSC) plot for the final reconstruction. The FSC=0.143 cut-off is marked as dashed line. The resolution limit for an FSC=0.143 is highlighted with an arrow. On the right, viewing direction distribution for particle projections. Heat map shows number of particles for each viewing angle.

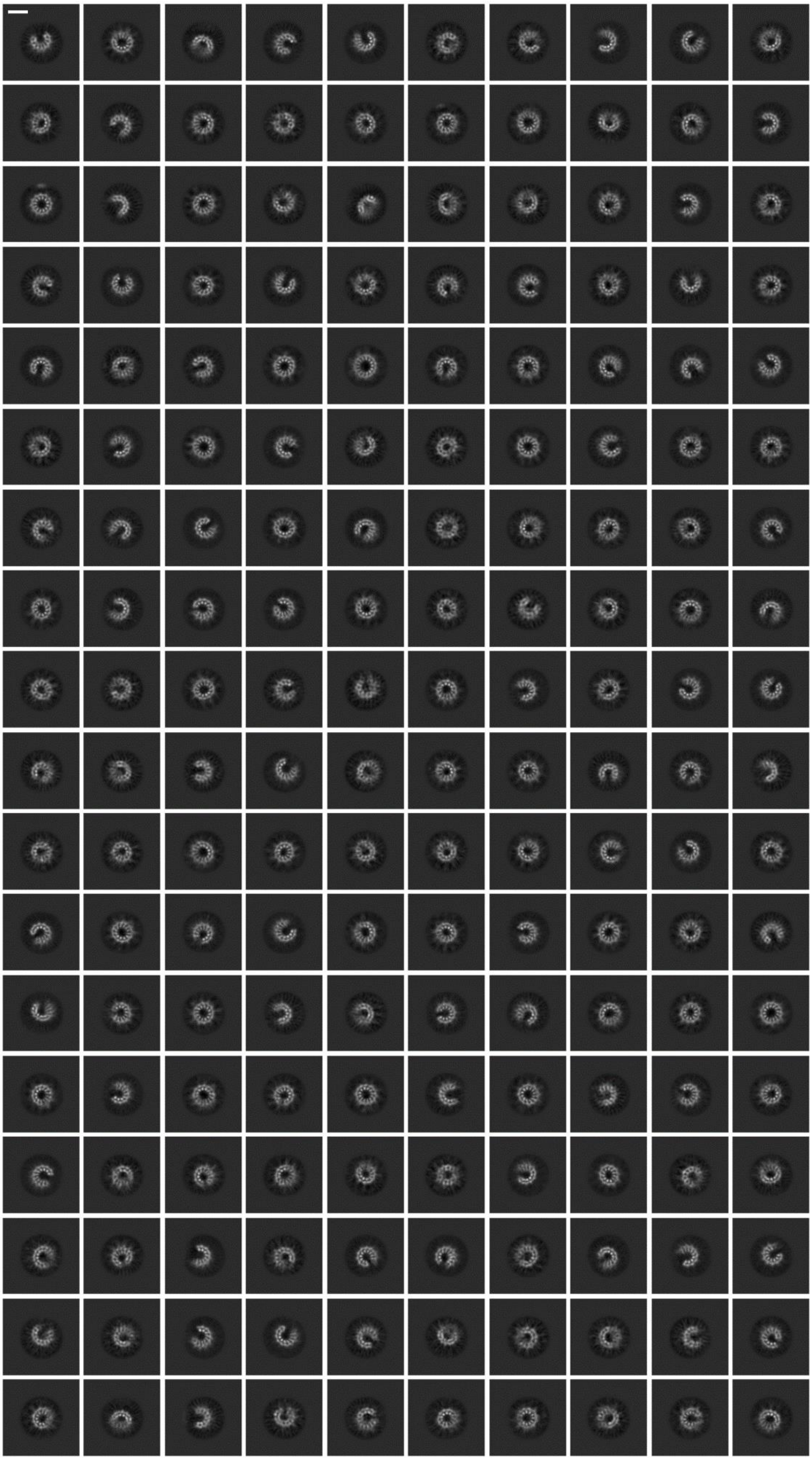

Supplementary Figure 2. Complete set of Cryo-EM 2D averages after the second 2D classification. The 2D class averages are ordered according to the number of particles averaged within the class. The scale bar inserted in the first class represents 6 nm.

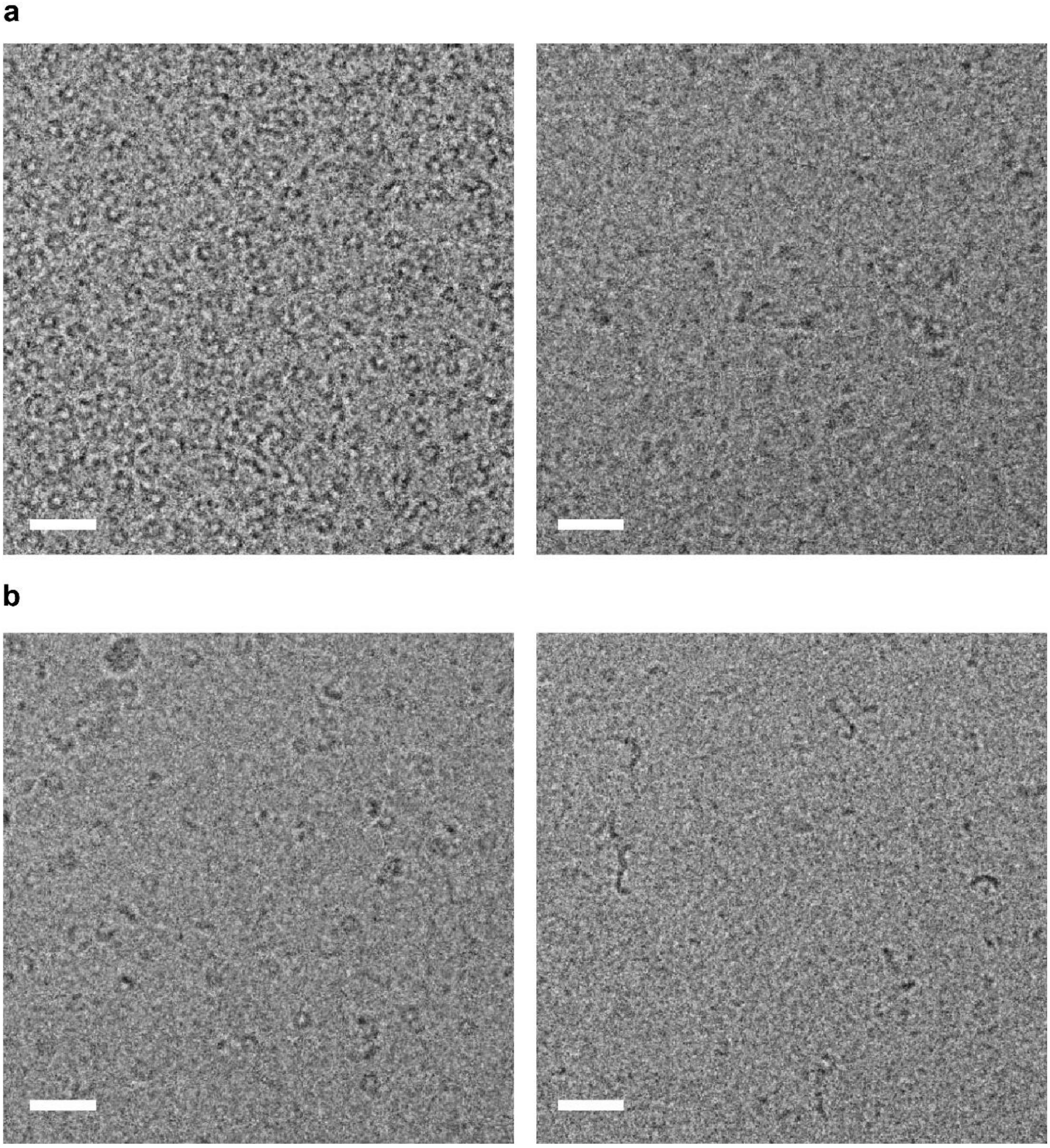

Supplementary Figure 3. Cryo-EM images of MucR and Ml5. (a) Images of wild-type MucR (left) and MucR^{L36L39I40A} (right). **(b)** Images of wild-type Ml5 (left) and Ml5^{L34L37I38A} (right). The white scale bars represent 20 nm.

```
MucR_Babortus     -MENLETNDESTELLLSLTADVVAAYVGNNSIRAGELPVLIAEVHAAFKRHVEREEAPVV
MucR_Bmelitensis  -MENLETNDESTELLLSLTADVVAAYVGNNSIRAGELPVLIAEVHAAFKRHVEREEAPVV
Ml2_Mloti         -MDIVETPSRNNDALIELTADVVAAYVSNNPVPVGELPNLISDVHAALGRVGGTAEQPPA
MucR_Smeliloti    --MTETSLGTSNELLVELTAEIVAAYVSNHVVPVAELPTLIADVHSALNNTTAPAPVVVP
Ros_Atumefaciens  --MTETAYGNAQDLLVELTADIVAAYVSNHVVPVTELPGLISDVHTALSGTSAPASVAVN
Ml1_Mloti         ---MTEEADKNIDTLIELTADVVSAYVSNNPVPVGDLPALIGQVHAALKGTAG-FVSAAK
Ml5_Mloti         ---MTEETESKADNLIELTAHVVSAYVSNNPVPVGELPGLIGQIHIALKGTAG-GAAPEK
Ml3_Mloti         ---MKELSNIEDKTVIELTADIVSAYVGNNPLPASGLPDLIASVSASVRKLAG--AVVVE
Ml4_Mloti         MPLRRKPLTDENINLIELTADIVSAYVSNNPVPVASLPDLIHSVNLSLSKVGR--PAEPE
MucR2_Ccrescentus --------MEDQSDLIEMTAGIVSAYVGNNVVSTADLPALIKQVHAALANVGAP-DAEAA
MucR1_Ccrescentus --------MEDKATLIELTAEIVANYVANNSTPVSELPALIRATHDALAGIGSPPAPTVE
                            ::.:** :*:  *  *. *: .    ** ** :.

MucR_Babortus     VEKPKPAVNPKKSVHDDYIVCLEDGKKFKSLKRHLVTHYNMTPEQYREKWDLDPNYPMVA
MucR_Bmelitensis  VEKPKPAVNPKKSVHDDYIVCLEDGKKFKSLKRHLVTHYNMTPEQYREKWDLDPNYPMVA
Ml2_Mloti         D-KQKPAVNPKRSVHDDYIVCLEDGKKFKSLKRHLMTHYDLTPDQYREKWNLDPSYPMVA
MucR_Smeliloti    VEKPKPAVSVRKSVQDDQITCLECGGTFKSLKRHLMTHHNLSPEEYRDKWDLPADYPMVA
Ros_Atumefaciens  VEKQKPAVSVRKSVQDDHIVCLECGGSFKSLKRHLTTHHSMTPEEYREKWDLPVDYPMVA
Ml1_Mloti         PEALEPAVPIRKSVTPDYIICLDDGKKFKSLKRHLSTHHGLTPDEYRAKWHLPADYPMVA
Ml5_Mloti         SEALKPAVPIRKSVTPDYIISLEDGKKFKSLKRHLATHYGLTPDEYRAKWELPADYPMVA
Ml3_Mloti         SPSLVPAVNPKKSVFPDYIICLEDGKKFKSLKRHLRTDYGLSPDDYRAKWGLPPDYPMVA
Ml4_Mloti         NPVLTPAVNPKKSVFPDYIVSLEDGRKFKSMKRHLG-LLGMTPDEYRTKWDLPRDYPMVA
MucR2_Ccrescentus PTPKEPAVPVKKSITPDYLICLEDGRKFKSLKRHLRTKYDMTPEDYRAKWGLPKDYPMVA
MucR1_Ccrescentus VVTKATPAQIRKSITPEALISFEDGKPYKTLKRHLT-THGMTVAEYKAKWGLPNDYPTTA
                        ... ::*: : : .:: * :  *::**** .:: :*:  *   * * . ** .*

MucR_Babortus     PNYAAARSRLAKKMGLGRKPKDA---------------
MucR_Bmelitensis  PNYAAARSRLAKKMGLGRKPKDA---------------
Ml2_Mloti         PNYAAARSQLAKKMGLGRKRKAR---------------
MucR_Smeliloti    PAYAEARSRLAKEMGLGQRRKRRGK-------------
Ros_Atumefaciens  PAYAEARSRLAKEMGLGQRRKANR--------------
Ml1_Mloti         PNYAAARSALAKTMGLGRKPKEPEARTRKKAAA-----
Ml5_Mloti         PNYAAARSALAKTMGLGRKPKEPETPAPAKRARKKAAA
Ml3_Mloti         PNYSATRSALAKSTGLGRKPAAAPAAVAKKGKAKA---
Ml4_Mloti         PNYAATRSALAKASGLGRKAAPVKKAPAKR-KAKA---
MucR2_Ccrescentus PNYAEARSNLAKQMGLGQGGRKPARKAK----------
MucR1_Ccrescentus PAYSEARSQMAKALGLGQGGRKGKTTRGRKG-------
                  * *: :** :**  ***:
```

Supplementary Figure 4. Sequence alignment (https://www.genome.jp/tools-bin/clustalw) of the highly investigated members of Ros/MucR family. Asterisks identify identical residues, dots similar residues. Uniprot accession numbers: Q2YRM3 for *B. abortus* MucR; Q8YFZ8 for *B. melitensis* MucR; Q985J6 for *M. loti* Ml2; Q04152 for *A. tumefaciens* Ros; Q989W1 for *M. loti* Ml1; Q98A76 for *M. loti* Ml5; Q984N1 for *M. loti* Ml3; Q98DI2 for *M. loti* Ml4; A0A0H3C684 for *C. crescentus* MucR2; A0A0H3C569 for *C. crescentus* MucR1.

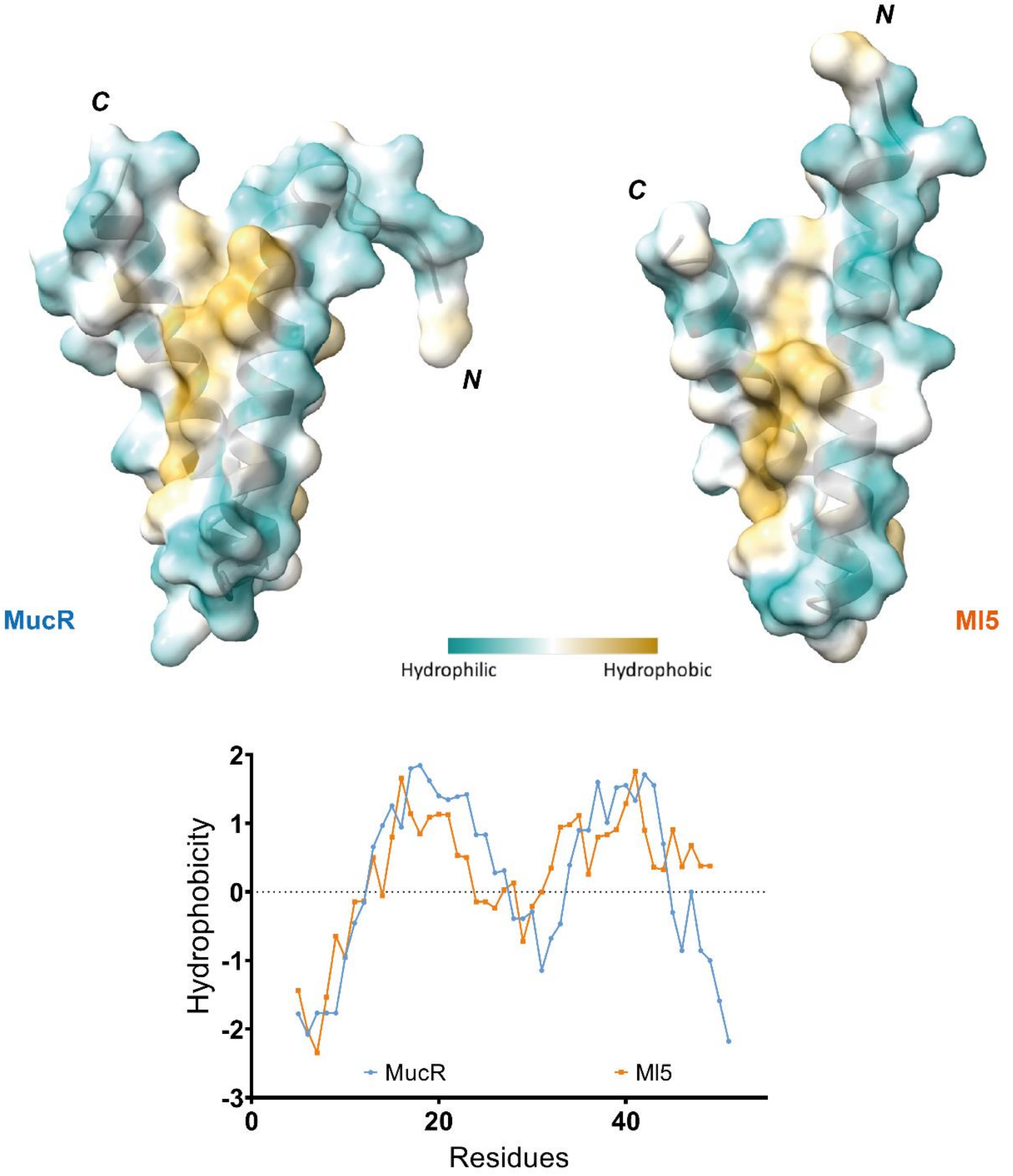

Supplementary Figure 5. Upper panel: MucR and Ml5 NTDs polarity surfaces. The hydrophobic surface outlined (built by Leu13, Leu15, Leu17, Ala19, Val21, Ala23, Ala24, Ala33, Leu36, Leu39, Ile40, Val43, and Ala46) is responsible for the α1/α2 interaction within each monomer and for inter-monomer compaction within the whole assembly of protein particles. The α1 and α2 helices are shown in cartoon representation below the transparent protein surface. Lower panel: MucR and Ml5 NTDs per-residue hydrophobicity score. Higher score means higher hydrophobicity, lower score indicates higher hydrophilicity.

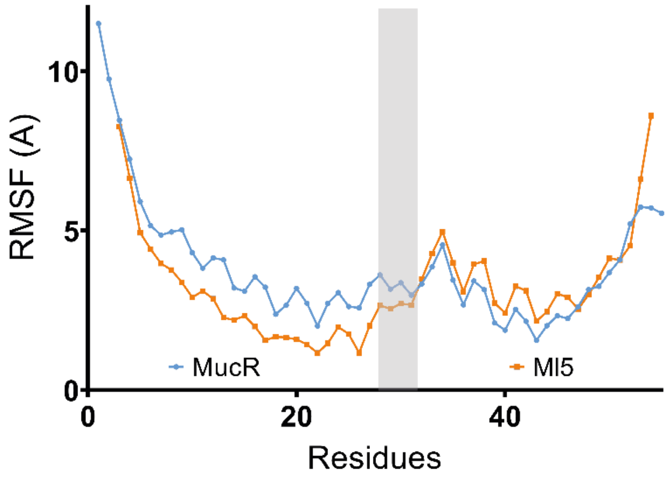

Supplementary Figure 6. MucR and Ml5 NTDs per-residue Cα Root Mean Square Fluctuations (RMSF) determined by NMSim software normal mode analysis. The grey rectangle indicates the linker region between α1 and α2 helices. The x-axis scale shows numbering according to the MucR sequence; for Ml5 residue numbering, see the alignment in Fig. 1B.

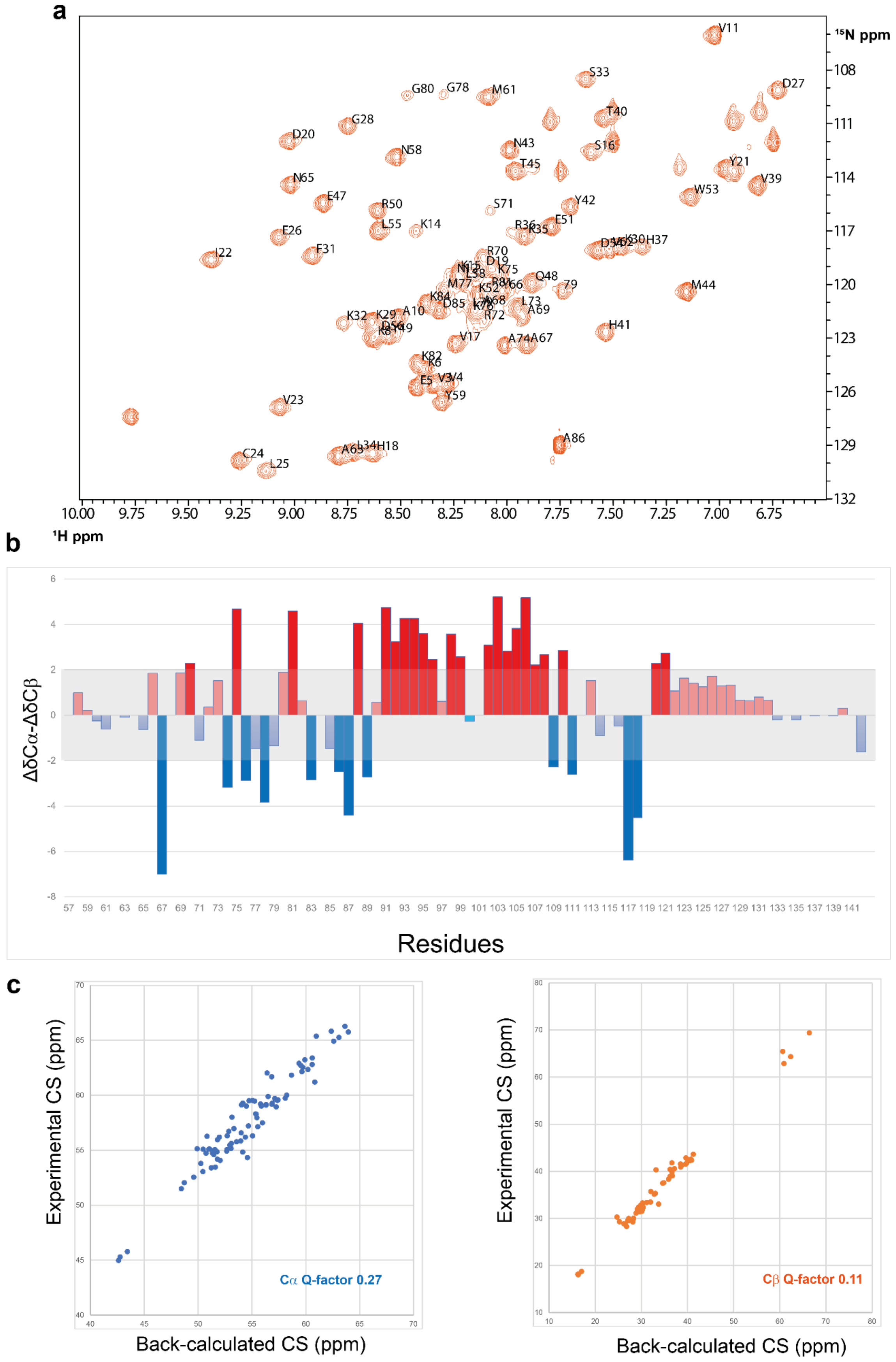

Supplementary Figure 7. (a) H_N-N assignment reported on the $MucR_{57\text{-}142}$ ^1H-^{15}N HSQC spectrum. **(b)** Per-residue ΔδCα-ΔδCβ for $MucR_{57\text{-}142}$. The cut-off values for the secondary structure elements are indicated by the

grey area. **(c)** Plot of experimental Cα (in blue) and Cβ (in orange) chemical shift correlation versus the same values back-calculated from the AF2 model. The calculated Q-factor is reported in the graph.

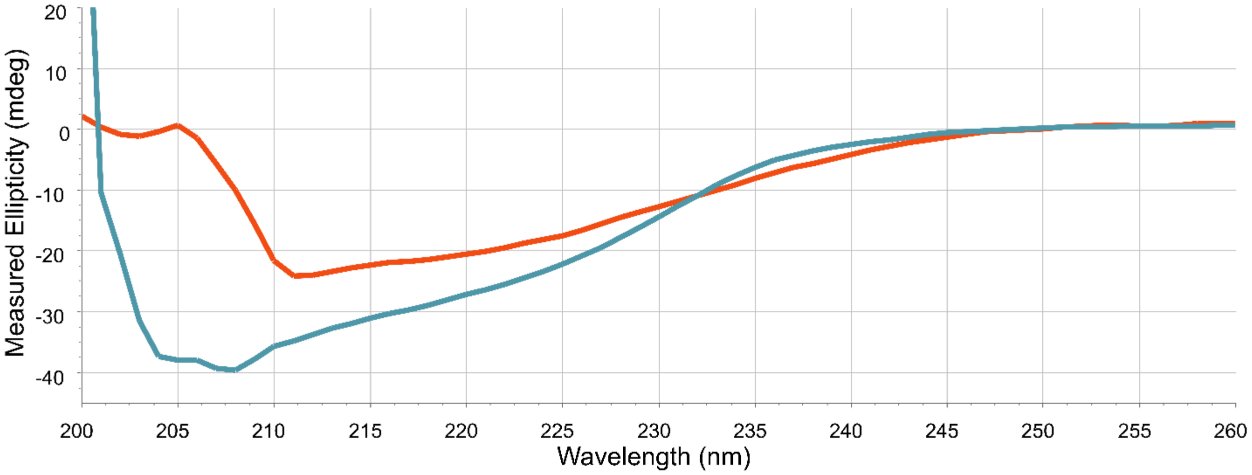

Supplementary Figure 8. Far-UV CD spectra of MucR$_{57\text{-}142}$ (in blue) and apo-MucR$_{57\text{-}142}$ (in orange).

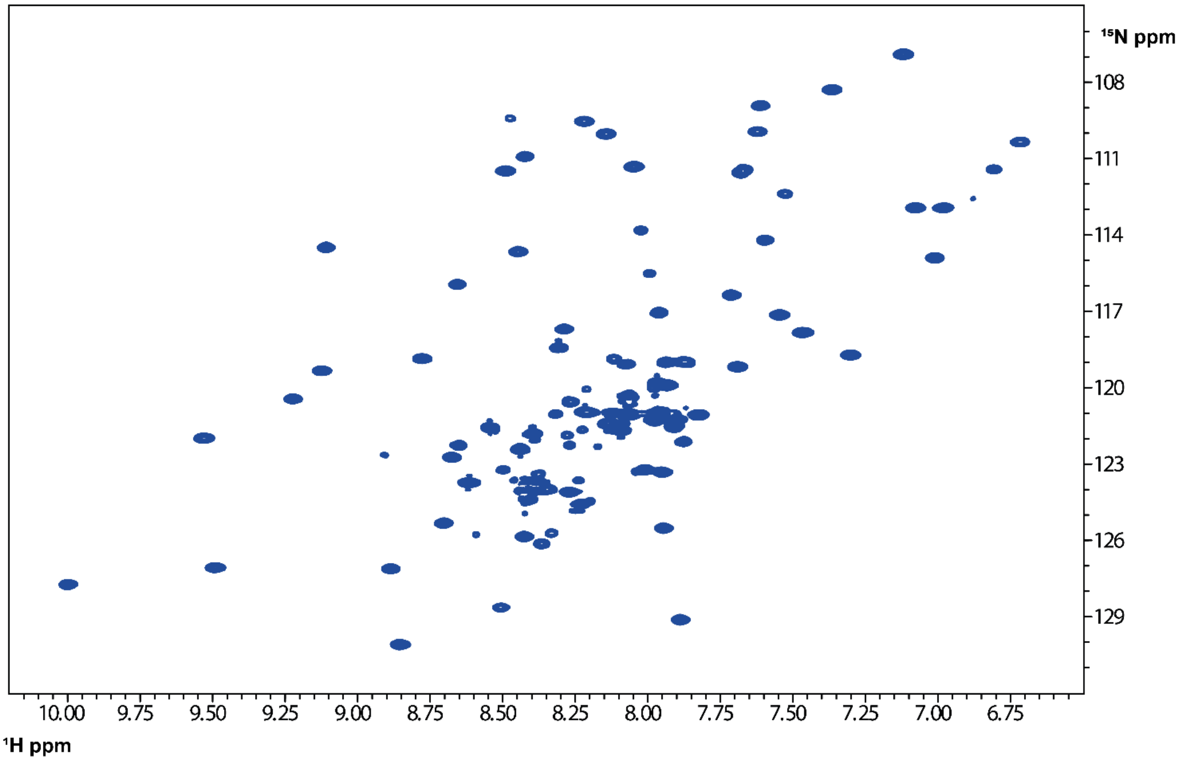

Supplementary Figure 9. ^{1}H-^{15}N HSQC spectrum of Ml5$_{56\text{-}154}$.

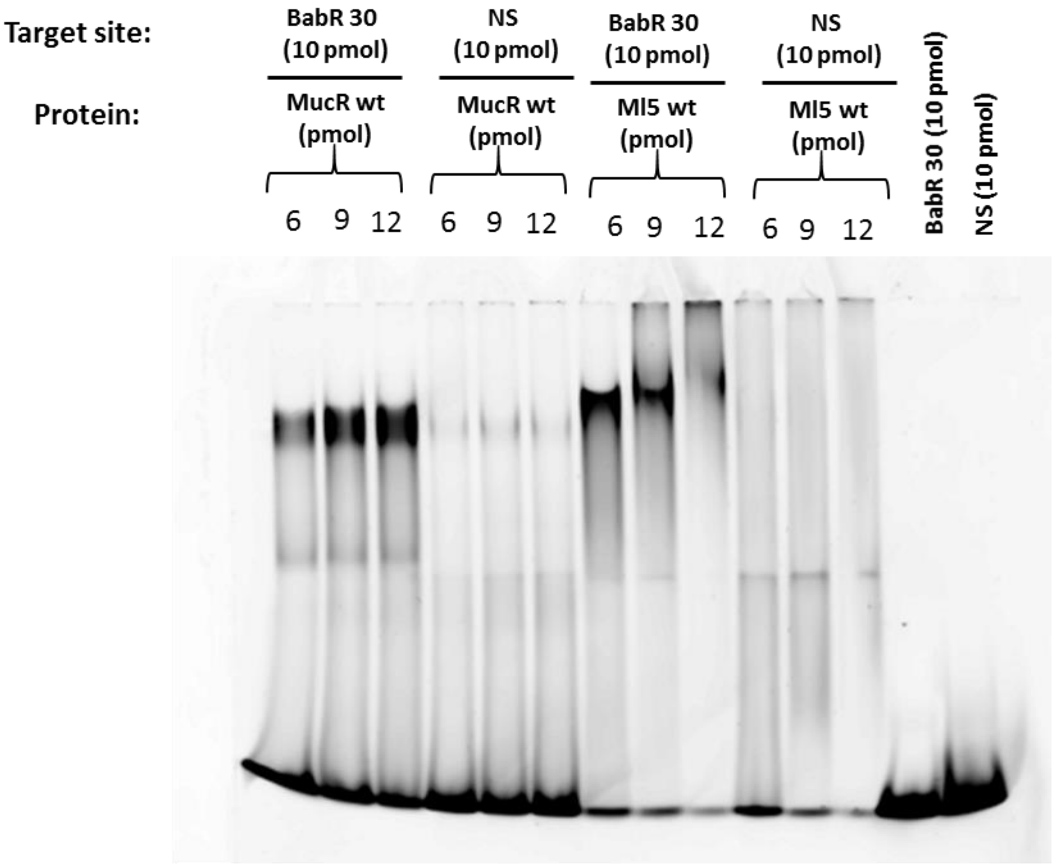


```
babR30: 5'-ATGAAGTTATATTCAATATAAAAGTAGAAT-3' 83 % AT
NS:     5'-CGCGGCACGACCGCAGCGGTCGGGTGGCAC-3' 20 % AT
```

Supplementary Figure 10: EMSA with MucR and Ml5. The sequences of the two DNA targets used and their AT content are indicated.

The sequence of babR30 is derived from the *babR* promoter(1); the NS oligonucleotide was designed as a scrambled GC-rich sequence.

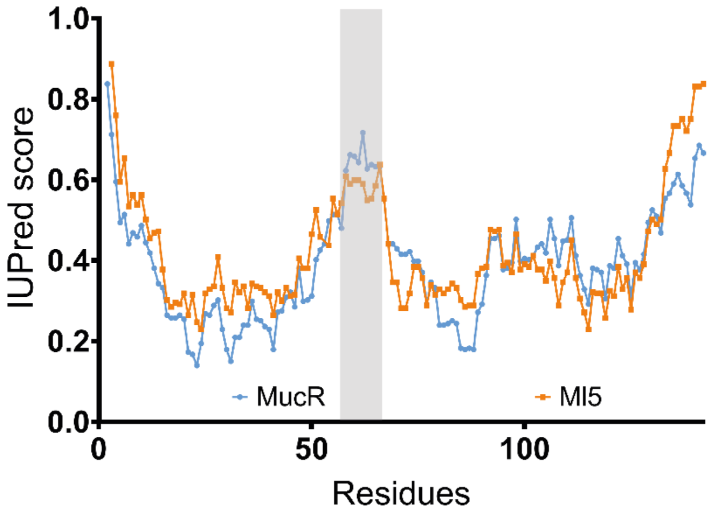

Supplementary Figure 11. Per-residue IUPred disorder propensity score of full-length MucR (in blue) and Ml5 (in orange). The grey rectangle indicates the linker region between the NTD and DBD. The x-axis scale shows numbering according to the MucR sequence; for Ml5 residue numbering, see the alignment in Fig. 1B.

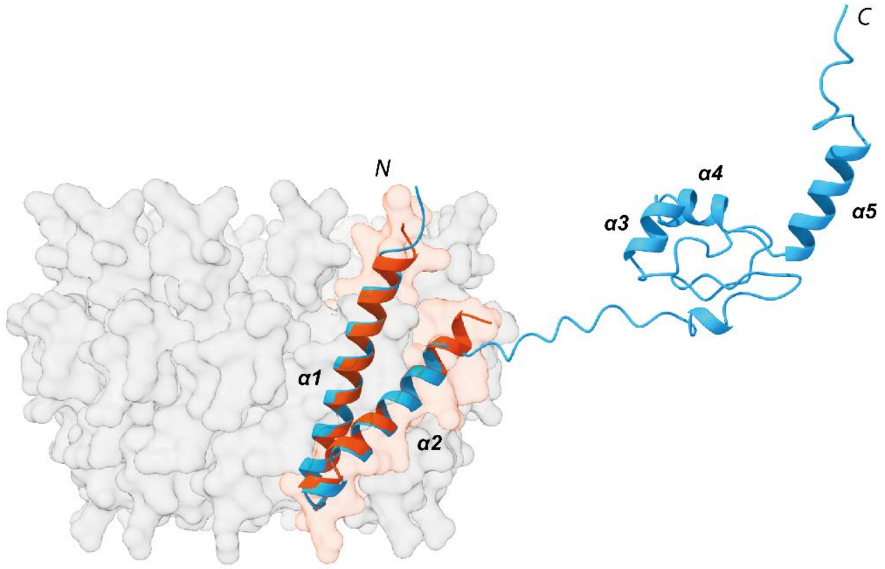

Supplementary Figure 12. Superimposition of one protomer of the AF2 model of the MucR NTD dodecameric complex (in red) with the AF model of full-length MucR AF model (in blue). The alignment was made with the Cα of residues 3-52 for both sequences giving an RMSD = 0.7Å.

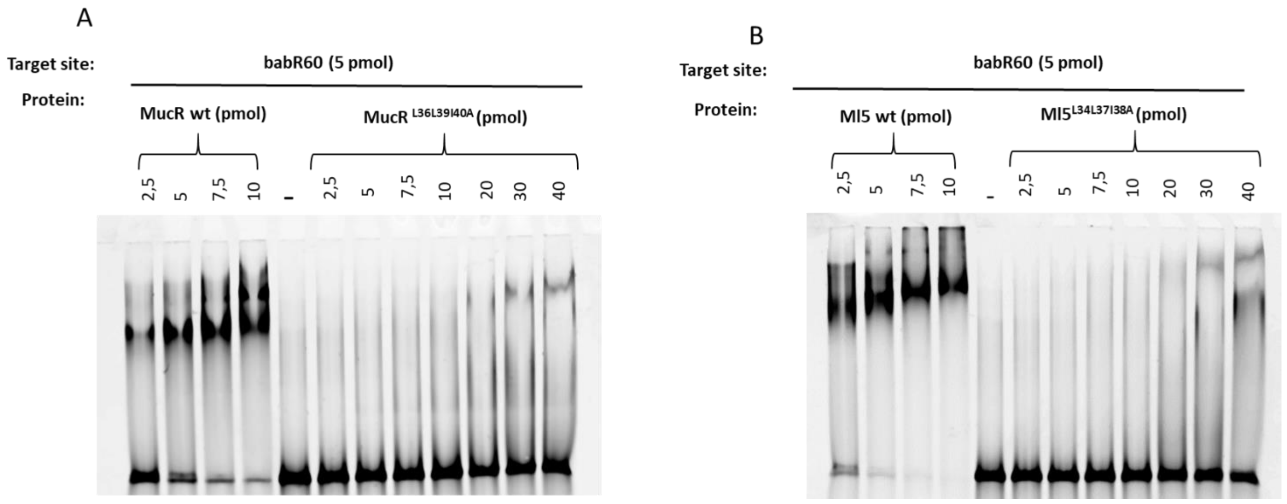

Supplementary Figure 13. EMSA of wild-type MucR, MucR^{L36L39I40A}, wild-type Ml5 and Ml5^{L34L37I38A} with the double stranded oligonucleotide babR60(1). The complexes of the two variant proteins with DNA are barely detectable.

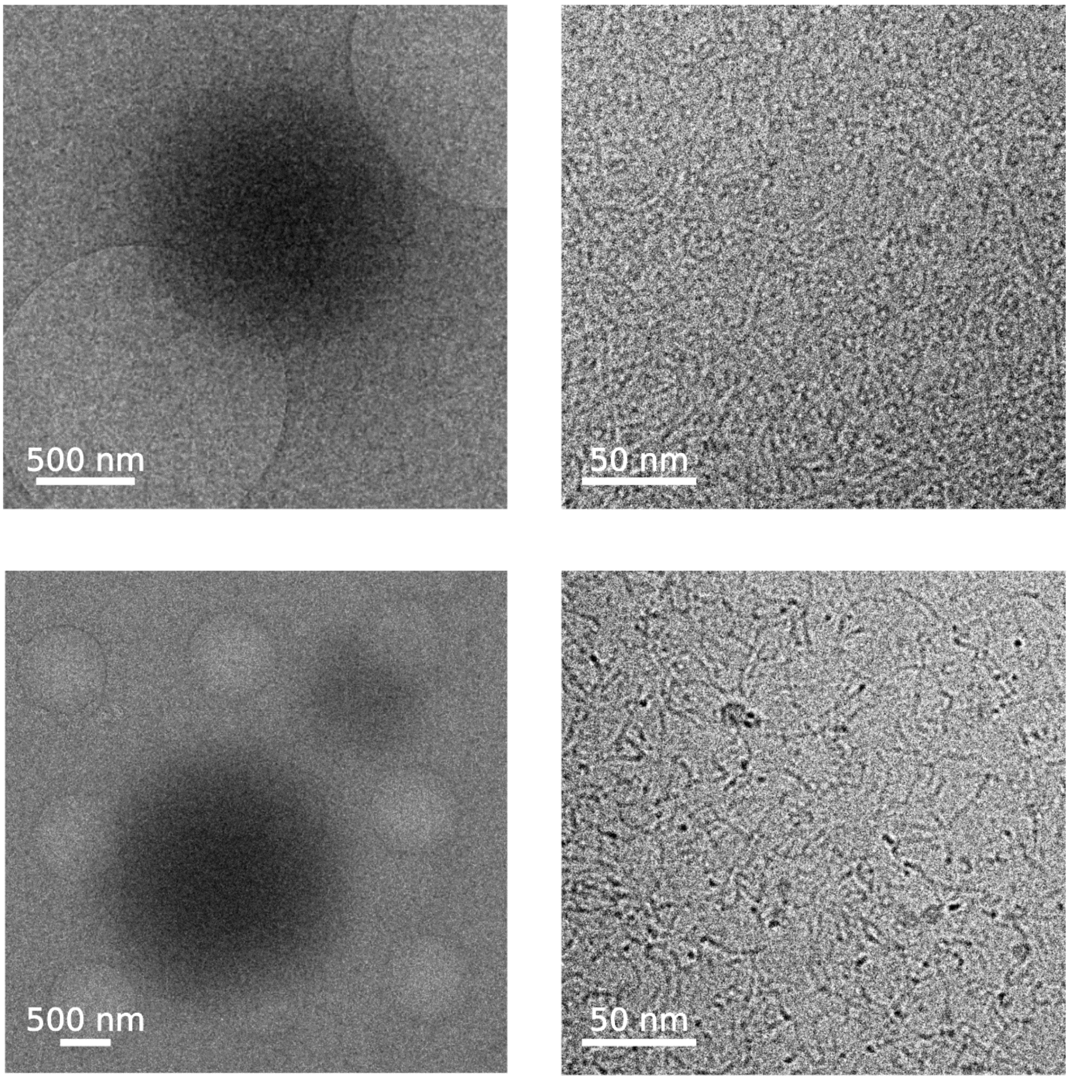

Supplementary Figure 14. Cryo-EM images of MucR-DNA complexes. Top: Images of MucR in complex with the 232 bp MucR promoter. Bottom: Images of MucR in complex with the 60 bp double stranded oligonucleotide babR60. Both complexes were imaged at low (6.700 x) and high magnification (73.000 x), left and right respectively.

References

1. Borriello, G., Russo, V., Paradiso, R., Riccardi, M.G., Criscuolo, D., Verde, G., Marasco, R., Pedone, P.V., Galiero, G. and Baglivo, I. (2020) Different Impacts of MucR Binding to the *babR* and *virB* Promoters on Gene Expression in *Brucella abortus* 2308. *Biomolecules*, **10**.
